# Virtual Reality Exergaming in Outpatient Stroke Rehabilitation: A Scoping Review and Clinician Roadmap

**DOI:** 10.3390/jcm14207227

**Published:** 2025-10-13

**Authors:** Błażej Cieślik

**Affiliations:** Healthcare Innovation Technology Lab, IRCCS San Camillo Hospital, 30126 Venice, Italy; blazej.cieslik@hsancamillo.it

**Keywords:** physiotherapy, serious games, post-stroke, telerehabilitation, physical activity, neurorehabilitation, motor recovery, Wii, Kinect, digital health

## Abstract

**Background/Objectives:** Outpatient stroke rehabilitation is expanding as inpatient episodes shorten. Virtual reality (VR) exergaming can extend practice and standardize progression, but setting-specific effectiveness and implementation factors remain unclear. This scoping review mapped VR exergaming in outpatient stroke care and identified technology typologies and functional outcomes. **Methods:** Guided by the JBI Manual and PRISMA-ScR, searches of MEDLINE, Embase, CENTRAL, Scopus, and Web of Science were conducted in April 2025. The study included adults post-stroke undergoing VR exergaming programs with movement tracking delivered in clinic-based outpatient or home-based outpatient settings. Interventions focused on functional rehabilitation using interactive VR. **Results:** Sixty-six studies met the criteria, forty-four clinic-based and twenty-two home-based. Serious games accounted for 65% of interventions and commercial exergames for 35%. Superiority on a prespecified functional endpoint was reported in 41% of trials, 29% showed within-group improvement only, and 30% found no between-group difference; effects were more consistent in supervised clinic programs than in home-based implementations. Signals were most consistent for commercial off-the-shelf and camera-based systems. Gloves or haptics and locomotor platforms were promising but less studied. Head-mounted display interventions showed mixed findings. Adherence was generally high, and adverse events were infrequent and mild. **Conclusions:** VR exergaming appears clinically viable for outpatient stroke rehabilitation, with the most consistent gains in supervised clinic-based programs; home-based effects are more variable and sensitive to dose and supervision. Future work should compare platform types by therapeutic goal; embed mechanistic measures; strengthen home delivery with dose control and remote supervision; and standardize the reporting of fidelity, adherence, and cost.

## 1. Introduction

Interest in virtual reality (VR) in healthcare continues to accelerate. Using a basic PubMed search for the term “virtual reality” run in late August 2025 with no filters, PubMed indexed 28,754 records for 2000–2024. The 10-year compound annual growth rate based on annual counts was higher in 2014–2024 (18.5%) than in 2000–2013 (13.3%). Burdea and Coiffet’s textbook, a foundational work in virtual reality, defines VR as a computer-generated simulation that creates a synthetic world responsive in real time [1]. In healthcare, VR is used to modulate symptoms during procedures, to deliver psychological interventions in controlled environments, and to support learning and rehearsal for clinicians and patients [2,3]. It also supports educational, planning, and communication tasks and is applied in perioperative care, chronic symptom management, oncology and palliative contexts, pediatric preparation, and selected assessment settings [4,5].

Within rehabilitation specifically, VR functions as a system-level framework that integrates hardware such as head-mounted displays, cameras, motion tracking, and haptics with software that renders interactive 3D tasks and logs performance, enabling immersive (head-mounted display, HMD) or non-immersive (screen or camera) task-oriented training [6]. Content typically falls into two streams: serious games, purpose-built to achieve clinical goals and progression, and commercial off-the-shelf games (COTS) repurposed for clinic or home for accessibility and motivation [7,8,9]. When a VR application adds a game-based exercise component that requires the user to move the body to interact, it is appropriately described as exergaming or active video gaming [10].

Globally, an estimated 93.8 million people were living with the effects of stroke in 2021, with 11.9 million incident strokes that year, and stroke remains a leading cause of death and disability worldwide [11]. In Europe, the annual economic burden is about EUR 60 billion, and rehabilitation is a major cost driver, accounting for roughly one-third of first-year post-stroke costs. Reported cost estimates include a mean rehabilitation cost of approximately EUR 3835 per patient during the acute episode and one-year rehabilitation costs of about EUR 33,500 for outpatient vs. about EUR 86,000 for inpatient programs (country specific) [12,13].

To help mitigate this burden, services increasingly use VR-supported rehabilitation as an adjunct to conventional therapy. Meta-reviews and the Cochrane update by Laver et al. (2025) report benefits for upper limb function, balance, and gait when VR is added to the usual care, with small but meaningful improvements in activity [14,15]. VR programs also target cognition, with systematic reviews in post-stroke cognitive impairment showing gains in cognitive tests and activities of daily living, although certainty varies, and psychological benefits include higher engagement and reductions in depressive symptoms in several meta-analyses [16,17,18]. Nevertheless, the cited reviews indicate that certainty remains limited, primarily due to small and largely single-center trials; heterogeneous interventions and comparators (content, dose, and supervision); diverse outcome measures; short follow-up; and persistent risks of bias (unclear randomization and allocation concealment, assessor blinding, and selective reporting) [14,15,17]. Consequently, certainty is typically rated low to moderate, with substantial heterogeneity and imprecision, especially for cognitive and mood outcomes [16,18].

Professional bodies highlight VR’s promise, particularly after COVID-19 accelerated implementation [19,20]. Prior to 2020, virtual stroke rehabilitation was used inconsistently; service disruptions during the pandemic prompted rapid scale-up. The American Physical Therapy Association notes that VR can tailor immersive rehabilitation for stroke, Parkinson disease, and multiple sclerosis [21]. Canadian stroke best practice recommendations endorse virtual rehabilitation as an alternative or adjunct to in-person therapy and advise offering it whenever in-person care is not feasible [19]. By contrast, the UK’s 2023 National Institute for Health and Care Excellence (NICE) stroke guideline characterizes VR games as relatively high cost and less common within telerehabilitation [22].

In practice, however, adoption remains limited. Surveys of therapists show only a minority use VR. For example, a Dutch physiotherapy survey found about 7% using VR for chronic pain rehabilitation [23]. In one study, 13% of clinicians believed older patients were familiar with exergames, and only 12% of patients recalled being advised to try them [24]. Multicenter scoping reviews have likewise reported that few studies have evaluated VR in routine care, and those that did often observed modest adherence, frequently around half of the planned sessions. Implementation barriers span clinician factors (limited familiarity, training needs, and unclear protocols); service factors (equipment costs, maintenance, and information technology support); and patient factors (comfort with technology, safety, and cybersickness) [20]. The literature consistently describes uptake as early and fragmented and identifies facilitators such as clear workflows, manageable setup time, remote monitoring, and lower-cost COTS options [25].

The aforementioned reviews and meta-analyses often pooled exergaming interventions across inpatient and outpatient settings. This scoping review aims to map and synthesize peer-reviewed experimental evidence on virtual reality exergaming for functional rehabilitation in outpatient stroke care, describing intervention characteristics, summarizing functional outcomes, and identifying contextual implementation barriers and facilitators.

## 2. Methods

### 2.1. Study Design

This scoping review followed JBI methodological guidance (Manual, Chapter 10.3) and best practice recommendations by Peters et al. [26]. Reporting adhered to PRISMA-ScR (checklist in Supplementary File S1). The protocol was registered a priori on OSF (https://osf.io/5kqde/, accessed on 10 October 2025). No critical appraisal was conducted, consistent with scoping review guidance and the protocol.

### 2.2. Eligibility Criteria

Eligibility criteria were defined a priori using the Population, Concept, and Context (PCC) framework [27]. Primary experimental studies enrolling adults post-stroke were eligible if they evaluated VR-based exergaming—interactive, game-like VR used to drive physical activity and motor practice—delivered in outpatient settings (clinic-based outpatient programs, community rehabilitation, or home-based outpatient care). Participants could be post-acute, subacute, or chronic, provided the intervention occurred after hospital discharge; early supported discharge or home programs were eligible if delivered as outpatient care. Eligible designs were randomized controlled trials (RCTs), quasi-randomized trials, non-randomized controlled studies, crossover trials, and single-group pre–post studies, published as full-text, peer-reviewed articles in English.

Studies were excluded if they were conducted in the acute inpatient phase (intervention delivered before hospital discharge) or in long-term residential care or if they enrolled mixed neurological samples without stroke-specific data. From the concept perspective, studies using VR solely for cognitive training, relaxation, or pain distraction were excluded, as were studies combining VR exergaming with robotics, functional electrical stimulation, or telerehabilitation when the independent effect of VR could not be isolated and development/engineering papers without an evaluative patient study. This avoids conflation with co-interventions and maintains interpretability of the synthesis within a PCC scoping review. From the context/outcomes perspective, studies without functional motor rehabilitation outcomes (e.g., cognitive-only and feasibility/acceptability/usability-only) were excluded. Reviews, meta-analyses, protocols without results, case reports/series, qualitative-only studies, cross-sectional surveys, editorials, and commentaries were excluded. Full-length peer-reviewed conference proceedings were eligible; abstracts without full papers were excluded.

### 2.3. Information Sources, Search Strategy, and Screening

Searches were conducted on 31 March 2025 in MEDLINE (via PubMed), Cochrane CENTRAL, Embase, Web of Science, and Scopus, from inception to the search date. The strategy followed the PCC framework: Population terms captured adults post-stroke; Concept terms reflected virtual reality and exergaming; Context terms addressed outpatient delivery and functional rehabilitation outcomes. Strategies were piloted and refined per database, using a vocabulary-plus-keyword approach (controlled terms such as MeSH/Emtree plus free-text synonyms). Full database-specific strings are provided in Supplementary File S2; the screening flow of full texts with exclusion reasons is detailed in Supplementary File S3. Backward and forward citation chaining of included studies were also performed.

Records were deduplicated using the Systematic Review Accelerator Deduplicator tool [28] and managed in Rayyan [29]. Title/abstract screening, retrieval of potentially eligible records, and full-text assessment were performed by a single reviewer (B.C.), with ambiguous cases resolved in consultation with an external expert. Grey literature and trial registries were not searched, as the objective was to map peer-reviewed experimental evidence.

### 2.4. Data Extraction and Synthesis

Data were extracted by a single reviewer (B.C.) using a piloted template. For each study, the main table captured VR system/platform, interaction modality, targeted motor domain, VR content type (serious vs. COTS), system type, outcomes, and key findings (Supplementary File S4, Supplementary Tables S1 and S2). A parallel implementation table charted barriers in four domains: recruitment/engagement, technical complexity, patient-related factors, and therapist/resource demands (Supplementary File S4, Supplementary Tables S3 and S4). Multiple reports of the same cohort were merged; missing items were coded as NI; ambiguities were resolved by re-review of the full texts.

Evidence was synthesized narratively in line with the PCC framework; meta-analysis was not attempted due to heterogeneity in design, outcomes, dose, and technology. An a priori typology coded immersion level (non-, semi-, or fully immersive); interaction modality (e.g., depth camera, balance board, sensor glove, inertial sensors, or HMD); delivery setting (clinic, home, or hybrid); therapeutic area (upper limb, lower limb, balance/gait, or multi-domain); and commercial availability (off-the-shelf or prototype). This scheme acknowledges the absence of a single standardized VR taxonomy in healthcare [6], aligns with established VR constructs (presence/immersion; reality–virtuality continuum) [1,30], and retains the common serious game vs. COTS distinction [7,8,9]. These codes underpinned the analytic results table; barrier themes were derived by inductive content analysis with constant comparison across studies.

## 3. Results

### 3.1. Search Results

**Figure 1:**
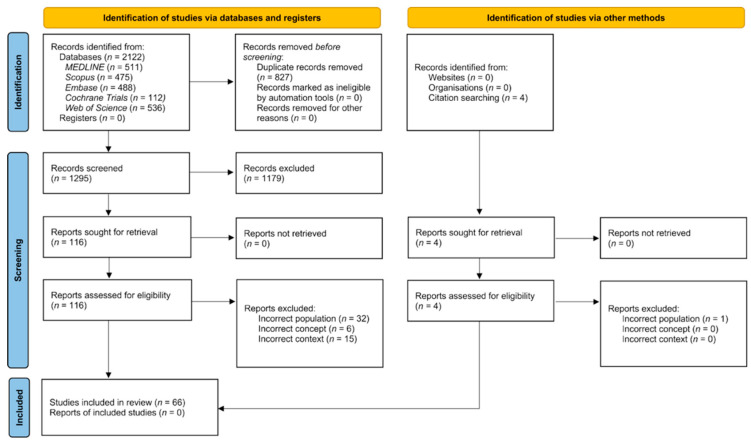
PRISMA flow diagram.

The database search identified 511 records from MEDLINE, 475 from Scopus, 488 from Embase, 112 from the Cochrane Central Register of Controlled Trials, and 536 from Web of Science. After deduplication, 1295 unique records were screened by title and abstract. Of 116 full texts, 63 met the criteria; citation chasing added three, for a final 66 studies (Figure 1). Reasons for exclusion at the full text stage are reported in Supplementary File S3.

Table 1 illustrates characteristics of the included studies. Across outpatient settings, 44 studies were clinic-based (67%), and 22 were home-based (33%). In clinics, serious games were slightly more common than commercial exergames (55% vs. 45%); designs were predominantly RCTs (75%), with fewer non-randomized controlled studies (9%) and single-group pre–post or longitudinal designs (16%). At home, most interventions used serious games (86%) rather than commercial exergames (14%); designs comprised RCTs (55%) and single-group pre–post/feasibility studies (45%). Overall, serious games accounted for 43 studies (65%) and commercial exergames for 23 (35%). By modality, across all trials, commercial console systems (COTS) were most common (35%), followed by camera-based systems (24%), hybrid setups (11%), gloves/haptics (11%), head-mounted displays (8%), IMU-based systems (8%), and locomotor platforms (5%).

### 3.2. Types of VR Interventions

The complete study inventory appears in Supplementary Files S4 (Supplementary Tables S1 and S2); technology categories are summarized in Table 2. Across the 66 included studies, interventions varied along three practical axes: immersion, interaction modality, and content.

Interventions varied by depth of immersion, ranging from non-immersive screens to semi-immersive setups and fully immersive HMD or CAVE environments. Non-immersive, screen-based programs were most often repurposed commercial consoles or camera capture for therapy, for example, Wii or Kinect titles used for balance and upper limb practice [72,73,74,83]. Semi-immersive systems added larger displays or haptics in rehab-specific suites, for instance, Jintronix and IREX in clinics or RIABLO with instrumented balance tasks [36,37,44,61]. Fully immersive approaches employed head-mounted displays, such as 360° mirror therapy and HMD-based task practice, and, in some cases, multi-screen CAVE or treadmill environments for gait [31,32,34,35,96].

The interaction modality defined how movement was sensed and fed back, spanning camera tracking, balance boards, wearable gloves, inertial sensors, haptic devices, and locomotor platforms. Studies used depth cameras for whole body or hand capture, balance boards for weight-shift tasks, sensor gloves for fine motor training, inertial sensors for postural control, and haptic styli for precise arm movements. Examples include Kinect body tracking and Wii Balance Board tasks, RAPAEL Smart Glove for dexterity, RIABLO with IMUs and a force platform, and a workbench system using a force feedback stylus [36,52,53,54,57,61,68,75,87].

Content and intent distinguished commercial exergames adapted for rehabilitation from purpose-built serious game platforms and determined whether tasks targeted upper limb dexterity, balance, gait, or multiple domains. Interventions clustered into commercial off-the-shelf programs and rehab-specific platforms with calibration, graded progression, and therapist dashboards. Illustrative examples include clinic-based Kinect or Wii protocols for balance and upper limb training, glove-based upper limb suites such as RAPAEL and Jintronix, immersive HMD programs, including mirror therapy, and CAVE or large screen treadmill scenes for community mobility practice [31,32,35,36,52,70,81,96].

### 3.3. Narrative Effectiveness Overview

**Figure 2:**
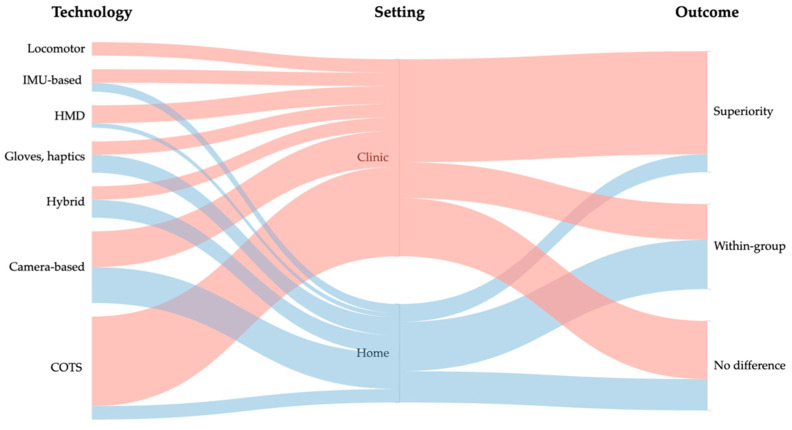
Alluvial map of outpatient VR exergaming trials by technology, setting, and outcome. Abbreviations: HMD: head-mounted display; COTS: commercial off-the-shelf; IMU: inertial measurement unit.

Effects were generally positive but heterogeneous. Across 66 trials, superiority on the prespecified primary functional endpoint was reported in 27 (41%), 19 (29%) showed within-group improvement only, and 20 (30%) found no between-group difference. Clinic-based studies more often achieved superiority (23/44; 52%) than home-based programs (4/22; 18%), whereas home studies more frequently reported within-group gains without outpacing the controls. By technology, patterns were mixed: COTS (10/23 superiority; 10/23 no difference), camera-based (7/16 superiority; 5/16 within-group), gloves/haptics (4/7 superiority), HMD (2/5 superiority), IMU-based (1/5 superiority; 3/5 within-group), hybrid (1/7 superiority; 5/7 within-group), and locomotor (2/3 superiority). Figure 2 visualizes these pathways in an alluvial map linking technology/setting/outcome; ribbon widths encode study counts, and colors encode setting, highlighting the clinic-heavy contribution to superiority outcomes. Detailed study-level data appear in Supplementary Files S4 (Supplementary Tables S1 and S2).

#### 3.3.1. Upper Limb

Multiple clinic and home programs reported greater improvements than the control in impairment or dexterity, for example, Fugl–Meyer Assessment Upper Extremity (FMA-UE), Motor Activity Log (MAL), Box and Block Test (BBT), or Wolf Motor Function Test (WMFT). Examples include Jintronix producing higher FMA-UE or MAL domains than usual care in clinic settings (*n* = 78 across two trials) [36,37] and a smart glove program yielding better BBT performance and grip strength alongside WMFT gains (*n* = 36) [52]. Immersive mirror therapy yielded larger improvements than conventional or classical mirror therapy on FMA-UE, MFT, and BBT (*n* = 45) [31], while a related HMD mirror therapy trial improved wrist subscores and BBT without a total FMA-UE difference (*n* = 52) [32]. Several studies were neutral or mixed: a custom motion controller protocol improved FMA-UE and WMFT within groups but did not beat the control, and the control arm outperformed on Instrumented Activities of Daily Living (IADL) and intrinsic motivation (*n* = 36) [63]; an immersive tracker-based system produced small, non-significant changes compared to conventional therapy (*n* = 18) [34]; home Wii exergaming did not outperform a home exercise program (*n* = 235) on the Action Research Arm Test (ARAT) [64]. In home programs, improvements were common but not universal: FMA-UE increased meaningfully in Leap Motion home training and in a glove-based home program, although some trials reported no between-group differences or effects confined to select outcomes [41,42,44,48,49,51,53,54,55,57,59,66,81,88].

#### 3.3.2. Lower Limb Balance and Gait

Several trials favored VR based on the Berg Balance Scale (BBS), Timed Up and Go (TUG), or related mobility metrics. Examples include greater BBS and faster 10-m walking with a custom stepping program (*n* = 20) [95]; significant advantages on BBS and TUG with Kinect or Wii protocols (Kinect corpus total *n* = 283; Wii corpus total *n* = 660) [72,73,74,83]; and better Motricity Index (MI), trunk control, and balance after an immersive HMD plus depth sensor program (*n* = 24) [35]. Large improvements were also reported in dedicated balance exergaming, for example, BBS increase of about five points and shorter TUG times after a balance board program (*n* = 6) [62]. Other studies were equivocal: Wii-based programs sometimes did not outperform controls on standardized balance tests (*n* = 84 across three trials) [68,70,85], and in a Kinect skiing protocol, Functional Reach Test (FRT) and TUG improved more in VR, while knee hyperextension angle and Barthel Index (BI) changed similarly across groups (*n* = 25) [84]. Treadmill or CAVE-style setups reported faster gait and better community mobility at follow-up (*n* = 20) [96]. Additional lower limb or multi-domain gains were observed in several studies, although between-group effects were not always consistent across outcomes or time points [40,43,45,61,65,67,94].

#### 3.3.3. Adherence and Acceptability

Adherence was generally good in supervised clinics and acceptable in most home programs, although dose attainment varied. Serious adverse events were not reported. Reported issues were mild and transient, such as brief dizziness or headache in immersive tracker systems or early session fatigue and occasional soreness, with rare session shortening for sensitivity to screen exposure [34,39,87]. Home programs occasionally noted eye strain or aches but no serious harm [60]. Several studies explicitly cited high enjoyment or motivation during exergaming and stable engagement over weeks [36,38,48,59,66].

### 3.4. Potential Barriers and Facilitators to Implementation

**Figure 3:**
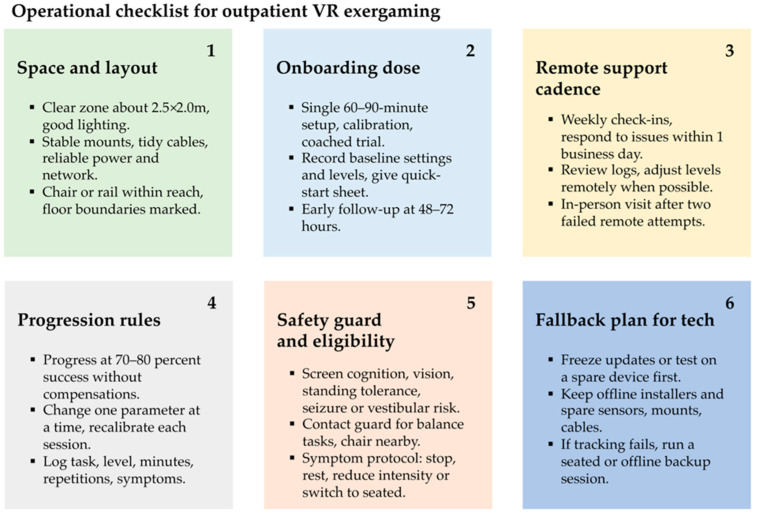
Minimum viable setup for outpatient VR exergaming. A six-item checklist derived from the barrier synthesis in Supplementary Tables S3 and S4.

Barriers and facilitators were synthesized into five themes across clinic- and home-based studies (Supplementary Files S4, Supplementary Tables S3 and S4). Barrier themes (recruitment/retention, technical complexity, patient factors, therapist/resources, and engagement/content) were translated into a six-item “minimum viable setup” checklist (space/layout, onboarding, remote support, progression, safety, and fallback), presented in Figure 3 as a pragmatic implementation aid.

Recruitment and retention emerged as persistent challenges across both clinic and home settings. Slow accrual, low consent, and mid-study withdrawals were frequently reported, often due to health events, transport or scheduling burdens, or competing commitments [56,58,65,79,82]. These patterns likely bias samples toward more mobile, less disabled outpatients.

Technical complexity often exceeded routine clinical and home workflows. Clinic deployments commonly required multi-device setups, calibration, and dedicated space, for example, cameras or IMUs with force platforms, HMD plus depth sensors, or Kinect with specific clearance and positioning [35,61,81,84]. Home programs reported software freezes, operating system (OS) updates breaking builds, Wi-Fi instability, tracking glitches, or cramped spaces, which triggered multiple tech support calls or in-person visits and sometimes extra hardware like mounts or adapters [38,48,49,51,56,60].

Eligibility criteria and patient factors narrowed who could use VR exergaming safely and effectively. Many studies excluded severe cognitive or visuospatial deficits and required minimum motor ability or standing tolerance, for example, Mini-Mental State Examination (MMSE) or Montreal Cognitive Assessment (MoCA) cut-offs, endurance thresholds, or device-specific ranges of motion for glove systems [40,53,54,68,69,74]. Even among eligible users, transient fatigue, soreness, dizziness, or headache occurred and were typically managed with rest or shorter sessions; some tasks provoked compensations or spasticity without coaching [34,39,75,87,94].

Therapist and resource demands were substantial in most implementations. Continuous or frequent therapist presence was often needed for setup, progression, safety guarding, and parameter tuning in clinics, with added minutes each session for game selection and calibration and support from assistants or students [35,37,45,66]. Home use still required onboarding visits, periodic check-ins, remote troubleshooting, and sometimes multiple home visits to keep systems running [38,49,53,60].

Engagement and content cut both ways for adherence and dose. Gamified feedback, scores, and visible progress often boosted motivation and practice time, and social or multiplayer features increased active movement and attendance [36,42,61,66]. Engagement dipped when games were too easy, repetitive, or poorly matched to ability and participants asked for more variety or progression; in one clinic trial, intrinsic motivation was lower with VR than with the control [56,60,63].

Several consistent facilitators helped the programs run smoothly and safely. Early hands-on onboarding, clear safety setups, responsive technical support, and caregiver or study partner involvement were repeatedly linked with better confidence, adherence, and troubleshooting, and therapist dashboards plus adaptive difficulty supported individualized progression [36,37,38,48,50,51,59,79].

## 4. Discussion

This scoping review mapped peer-reviewed experimental evidence on VR exergaming for functional rehabilitation in outpatient stroke care and offers a clinician-oriented roadmap. Across studies, functional gains appear achievable, most consistently in supervised clinic programs targeting upper limb function and balance or gait, whereas home-based implementations often yield within-group gains but less often outperform active comparators. Evidence is most consistent for COTS and camera-based systems. Glove or haptic interfaces and locomotor platforms are promising but less studied. HMDs yield heterogeneous results. Adherence is generally good; adverse events are infrequent and transient; and real-world impact seems contingent on dose control, tele-supervision, and manageable setup demands.

Situated within a heterogeneous evidence base, our outpatient-focused synthesis makes a specific observation: many reviews and meta-analyses pool trials across inpatient wards, outpatient clinics, and home or community settings, which blurs setting-specific conclusions [14,97]. Within that mixed body of work, balance and upper limb signals are the most consistent. Wii Fit-style and other exergame programs commonly improve BBS and TUG, with weaker or null effects on gait speed, and adverse events are typically transient, patterns echoed by umbrella summaries that also highlight considerable methodological heterogeneity [98,99]. Modality may matter. A recent network meta-analysis ranked head-mounted immersive VR above non-immersive systems such as Kinect or Wii for upper extremity outcomes, although superiority is not universal and appears to depend on dose and comparators [100]. At the same time, activity and participation outcomes remain mixed. Cochrane updates report small benefits for ADL when VR augments the usual care but limited or uncertain effects for gait speed, participation, and quality of life, underscoring a gap between impairment-level gains and real-world transfer [14]. A further blind spot is designed for sustained engagement. Narrative work notes that diegesis and purpose-driven tasks, elements likely to influence motivation and delivered dose, are rarely specified or measured [101]. Coherent in-world context and patient-prioritized goals can support autonomous motivation and adherence, as predicted by self-determination theory, which is central to sustaining use over weeks rather than sessions [102]. In stroke VR exergaming, diegetic framing and meaningful progression have been proposed as levers to enhance engagement and dose [101]. Clinically, selecting or configuring programs with clear roles, immediate feedback, and visible goal progression can help maintain adherence, a prerequisite for long-term benefit in chronic rehabilitation [103]. In this context, our review adds outpatient-specific nuance: supervised clinic exergaming more often achieves between-group superiority than home-only implementations, which frequently show within-group gains without clear advantages over active controls, a pattern that helps to reconcile divergences in the pooled literature.

### 4.1. Bigger Picture

VR exergaming effectiveness lies mainly in its mechanisms. It is designed to deliver high-frequency, task-specific augmented feedback and adaptive practice that are difficult to sustain in one-to-one physiotherapy, operationalizing core motor learning principles such as variable practice, graded challenge, and timely knowledge of performance and results [104,105]. Camera-based systems can provide real-time kinematic cues and error augmentation to refine trajectories, glove and haptic interfaces add force and proprioceptive signals to shape grasp–release and coordination, and IMU-based wearables extend feedback into the home, enabling live or asynchronous coaching and objective exposure tracking [106,107,108]. VR environments also support dual-task training by safely layering cognitive demands onto locomotor tasks, which can reduce cognitive–motor interference and improve gait metrics in stroke cohorts in the short term [109,110]. Downstream transfer to activities of daily living and quality of life is less consistent, which underscores the need to link these mechanisms to durable functional change.

Economics and workload also shape real-world effectiveness. Staff time scales with device complexity and affects dose, so supervised acclimatization, check-ins, and remote support are part of the intervention rather than overhead [111]. Evidence on costs is limited and methodologically mixed. Reviews of VR or digital motor rehabilitation report relatively few full economic evaluations and heterogeneous methods, and in some reports, VR-based telerehabilitation appears less costly than clinic delivery, although estimates depend on perspective, time horizon, and what costs are included [112,113]. One evidence review noted per-participant savings for a balance program, but this was context-specific and driven by model assumptions, so it should not be generalized [114]. In our sample, beyond COTS platforms, only 9 out of 37 purpose-built serious game devices were commercially available. Limited availability constrains price transparency, maintenance and replacement planning, and complicates cost or budget impact analyses. These factors may contribute to the pragmatic appeal of COTS in outpatient services, where acquisition and procurement are simpler, but the net cost-effectiveness relative to purpose-built systems remains uncertain. Implementation studies also highlight device choice, training time, and workflow fit as key barriers or facilitators, suggesting that manageable setup demands are a determinant of effectiveness rather than a convenience [115].

### 4.2. Clinical Implications and Future Studies Directions

VR exergaming may serve as a practical adjunct to extend practice, support more structured progression, and help maintain continuity of care across outpatient pathways, particularly as Early Supported Discharge (ESD) becomes more common and inpatient episodes shorten [116,117]. However, evidence for transfer to activities of daily living, quality of life, and long-term effects remains mixed. It may be considered for adults post-stroke who meet basic safety and cognitive–visual–motor criteria, for example, adequate attention, absence of severe neglect, sufficient vision, standing tolerance, and task-specific range of motion. This is especially appropriate for motor–cognitive or dual-task exergaming, which can improve gait, balance, and some cognitive measures, though ADL and quality of life effects are inconsistent [118]. By contrast, for individuals with severe cognitive or motor deficits, who are largely excluded from current trials [14], feasibility and net benefit are uncertain. If attempted, programs should prioritize safety, caregiver involvement, simplified interaction, and realistic goals, recognizing that gains may be smaller and adherence more variable. A staged approach can help, starting with supervised clinic sessions to establish calibration, technique, and early gains, then moving to home or tele-supervised sessions to increase dose when safe and feasible. Platform choice can be aligned with the therapeutic goal, for example, camera-based, glove, or haptic systems and immersive mirror therapy variants for upper limb function and dexterity and commercial balance boards or purpose-built locomotor environments for balance and gait. A step-up or step-down service model may be appropriate, positioning VR exergaming as an adjunct to the usual care rather than a replacement unless local protocols and resources support standalone use.

Future work could compare platform typologies aligned to therapeutic goals, for example, immersive HMD mirror training vs. semi-immersive camera or screen systems for upper limbs and commercial balance boards vs. purpose-built locomotor environments for gait and balance. Studies should be stratified by impairment severity, time post-stroke, cognitive status, and presence of neglect to identify likely responders. From a cost perspective, it is timely to test current generation consoles, as platforms like Nintendo Switch^®^ offer distinct feedback channels and have shown promising outcomes in other neurological populations [119,120]. Mechanistic evaluation can be embedded, using kinematics, movement smoothness, error signals, and engagement metrics to test motor learning pathways that link practice to functional change. Studies would benefit from inclusive designs that enroll older adults with low digital literacy and rural participants alongside loaner hardware and support models to address access barriers. Personalization can be examined through AI-driven adaptive algorithms that tune task parameters to individual learning curves and patient-prioritized goals, with pre-specified minimal clinically important differences guiding adaptation and decision rules [121].

### 4.3. Limitations

Several limitations should be noted. This scoping review was designed to map rather than pool effects, so no meta-analysis was undertaken, and comparative efficacy could not be established. Study selection and data extraction were performed by a single reviewer, with consultation to resolve ambiguities. The search was limited to peer-reviewed English language sources and did not include a dedicated grey literature sweep, so relevant non-English or non-indexed evidence may have been missed. This approach may increase the risk of misclassification and of omitting eligible studies; although calibrated single-reviewer screening conducted by experienced researchers can have low omission rates, it remains a limitation [122]. Future updates should incorporate dual independent processes or reliability checks. The evidence base is highly heterogeneous in interventions, comparators, dosing, chronicity, and outcomes, and the three-category outcome coding necessarily simplifies this variability and may obscure nuance. Generalizability is limited, because many trials excluded individuals with severe cognitive or motor impairments and were conducted in well-resourced outpatient services. Sample sizes were often small with short follow-up, particularly in home programs, constraining inferences about precision and durability. Recent benchmarks indicate that, to detect a medium effect at 80% power requires ~126 participants in total, while a large effect requires ~26 [123]. Most included RCTs enrolled ≤60 participants (many ≤30), with only a few nearing ~100; thus, many were underpowered for medium effects—and some even for large effects—so null or mixed results should be interpreted cautiously. Technology drift is likely: across studies, hardware and software evolved (e.g., legacy Wii/Kinect vs. contemporary HMDs with inside-out tracking), changing tracking fidelity, latency, field of view, cybersickness risk, setup burden, and available content. Several platforms are now discontinued or run on deprecated OSs, and routine updates can alter interaction/performance mid-study. These shifts reduce cross-study comparability and limit the generalizability of older findings to current products. Finally, the barrier and facilitator synthesis relied on authors’ reported statements rather than direct observation and used descriptive counts rather than inferential analysis.

## 5. Conclusions

Technology-mediated rehabilitation is moving into outpatient and home care as stroke pathways shorten and inpatient dose contracts. VR exergaming has risen in prominence over the past decade, and changing health policies will further test its delivery. Based on a body of predominantly small, single-center trials with heterogeneous interventions and short follow-up, overall certainty of effects is best characterized as low to moderate. In this review, outpatient evidence indicates functional benefits of this modality, most consistently in supervised clinic-based programs, notably for upper limb function and balance or gait. Home-based implementations often deliver within-group gains but less frequently outperform active comparators, indicating sensitivity to dose, comparator intensity, and technology maturity. Signals are strongest for commercial off-the-shelf and camera-based systems; gloves or haptics and locomotor platforms are promising but less extensively studied, and head-mounted display interventions show mixed findings. Adherence is generally good, and adverse events are infrequent and transient. Durability beyond the intervention period remains uncertain: few studies include longer-term follow-up, activity/participation and quality-of-life outcomes are variably reported, and maintenance of gains is inconsistent.

Implementation constraints remain prominent and likely shape delivered dose and observed outcomes. Recurring issues include recruitment and retention, technical complexity, patient selection and safety needs, therapist and resource load, and alignment of game content with patient ability. Overall, VR exergaming appears clinically viable but operationally contingent. Priorities include strengthening home-based delivery through clearer dose control and tele-supervision, standardizing progression and safety procedures, and reducing technical friction with simpler setup and responsive support, alongside routine reporting of fidelity, adherence, and cost to improve effectiveness and scalability. In practice, a pragmatic pathway is to begin with supervised clinic onboarding for calibration and safety, then transition to tele-supervised home blocks with explicit weekly dose targets while routinely tracking fidelity, adherence, adverse events, and basic costs.

## Figures and Tables

**Table 1:** Characteristics of included studies.

Characteristics of Studies	*n* (%)
**Publication year**	
2025–2021	27 (41)
2020–2016	26 (39)
2015–2011	10 (15)
≤2010	3 (5)
**Design**	
RCTs	45 (68)
Non-randomized controlled studies	4 (6)
Single-group pre–post	17 (26)
**Type of VR**	
Serious exergame	43 (65)
Commercial exergame	23 (35)
**Environment**	
Clinic-based outpatient	44 (67)
Home-based outpatient	22 (33)
**Experimental intervention**	
HMD	5 (8)
Camera-based	16 (24)
Gloves, haptics	7 (11)
IMU-based systems	5 (8)
COTS	23 (35)
Hybrid	7 (11)
Locomotor	3 (5)

RCTs: randomized controlled trials; VR: virtual reality; HMD: head-mounted display; IMU: inertial measurement unit; COTS: commercial off-the-shelf.

**Table 2:** Types of devices and technology used within VR interventions.

Category	Technology	Area	Setting	CA	Sample Size	References
**HMD**	HMD VR mirror therapy	Upper limb	Clinic	No	45	[31]
	HMD VR mirror therapy + Leap Motion,	Upper limb	Clinic	No	52	[32]
	Pico Neo 2 HMD + REHAGO software	Upper limb	Home	No	48	[33]
	Custom immersive VR HMD with IMU	Upper limb	Clinic	No	18	[34]
	Oculus Quest 2 HMD + Kinect sensor	Multiple	Clinic	No	24	[35]
**Camera-based**	Jintronix (Kinect)	Upper limb	Both	Yes	98	[36,37,38]
	Kinect2Scratch (custom PC games)	Upper limb	Clinic	No	18	[39]
	Motion Rehab AVE 3D (Kinect + projection)	Upper limb	Clinic	No	31	[40]
	PIXER home (Kinect v2)	Upper limb	Home	No	10	[41]
	VERGE tele-exergame (Kinect)	Upper limb	Home	No	20	[42]
	Stomp Joy	Lower limb	Clinic	No	22	[43]
	GestureTek IREX	Upper limb	Clinic	Yes	36	[44,45]
	Kinect Rapid Movement Training platform	Lower limb	Both	No	30	[46]
	Kinect v2 home guidance system	Upper limb	Home	No	12	[47]
	HoVRS, home platform using Leap Motion	Upper limb	Home	No	43	[48,49]
	HEAD platform (Kinect plus Leap Motion)	Upper limb	Home	No	34	[50]
	EvolvRehab Body (Kinect v2 plus Leap Motion)	Upper limb	Home	Yes	8	[51]
**Gloves, haptics**	RAPAEL or Neofect Smart Glove	Upper limb	Both	Yes	70	[52,53,54]
	MusicGlove (Flint Rehab)	Upper limb	Home	Yes	17	[55]
	YouGrabber sensor gloves (YouRehab)	Upper limb	Clinic	No	12	[56]
	Workbench VR with Phantom Omni stylus	Upper limb	Clinic	No	22	[57]
	Virtual Glove (infrared glove)	Upper limb	Home	No	27	[58]
**IMU-based systems**	ArmeoSenso (home)	Upper limb	Home	Yes	11	[59]
	Neurofenix NeuroBall/NeuroBands	Upper limb	Home	Yes	30	[60]
	RIABLO (wireless IMUs + force platform)	Lower limb	Clinic	Yes	15	[61]
	myBalance IMU balance board (custom)	Lower limb	Clinic	No	6	[62]
	PC games with CyWee Z motion controller	Upper limb	Clinic	No	36	[63]
**COTS**	Nintendo Wii/Wii Fit	Multiple	Both	Yes	660	[64,65,66,67,68,69,70,71,72,73,74,75,76,77,78]
	PlayStation 2 EyeToy	Multiple	Clinic	Yes	68	[66,76,79]
	Xbox 360 Kinect	Multiple	Both	Yes	283	[79,80,81,82,83,84,85,86]
**Hybrid**	MindMotion PRO (motion camera + wrist IMUs)	Upper limb	Clinic	Yes	10	[87]
	Rehabilitation Gaming System (Eodyne)	Upper limb	Home	No	35	[88]
	Custom home gaming system	Upper limb	Home	No	11	[89]
	Recovery Rapids: Kinect + glove	Upper limb	Home	No	14	[90]
	Dual 3D displays with PneuGlove, IMUs	Upper limb	Clinic	No	6	[91]
	Passive hand orthosis, SaeboMAS software	Upper limb	Home	No	19	[92]
	LANR platform (Kinect plus Leap Motion)	Upper limb	Home	No	9	[93]
**Locomotor**	VR cycling system (recumbent bike + screen VR)	Lower limb	Clinic	No	8	[94]
	VR stepping exercise (screen-based)	Balance	Clinic	No	20	[95]
	CAVE-style VR treadmill	Lower limb	Clinic	No	20	[96]

CA: commercial availability; VR: virtual reality; HMD: head-mounted display; IMU: inertial measurement unit; COTS: commercial off-the-shelf; PC: personal computer.

## Data Availability

All data generated and analyzed during this review are included in this manuscript and its Supplementary Materials.

## Supplementary Materials

The following supporting information can be downloaded at https://www.mdpi.com/article/10.3390/jcm14207227/s1: Supplementary File S1: PRISMA checklist; Supplementary File S2: Search strategy for databases; Supplementary File S3: List of excluded studies with reasons; Supplementary File S4: Data extraction tables.
