# Supplementary material for: Virtual Reality Exergaming in Outpatient Stroke Rehabilitation: A Scoping Review and Clinician Roadmap
Source: J Clin Med. 2025 Oct 13;14(20):7227. doi: 10.3390/jcm14207227 (PMC12565396; doi:10.3390/jcm14207227)
Supplement: Supplementary file 1 [file jcm-14-07227-s001.zip › Supplementary File S2.pdf]

Supplementary File 2. Search strategy for databases

Database	Search string
**Medline (via PubMed)**	("stroke"[MeSH Terms] OR "Brain Ischemia"[MeSH Terms] OR "Cerebral Hemorrhage"[MeSH Terms] OR "Intracranial Thrombosis"[MeSH Terms] OR "stroke"[Title/Abstract] OR "post-stroke"[Title/Abstract] OR "ischemic stroke"[Title/Abstract] OR "hemorrhagic stroke"[Title/Abstract] OR "cerebrovascular accident"[Title/Abstract] OR "brain infarction"[Title/Abstract] OR "CVA"[Title/Abstract] OR "transient ischemic attack"[Title/Abstract] OR "Brain Ischemia"[Title/Abstract] OR "intracranial hemorrhage"[Title/Abstract] OR "cryptogenic stroke"[Title/Abstract]) AND ("Virtual Reality"[MeSH Terms] OR "Exergaming"[MeSH Terms] OR "Virtual Reality"[Title/Abstract] OR "VR"[Title/Abstract] OR "virtual environment*"[Title/Abstract] OR "virtual rehabilitation"[Title/Abstract] OR "exergam*"[Title/Abstract] OR "active video game*"[Title/Abstract] OR "interactive game*"[Title/Abstract] OR "serious game*"[Title/Abstract] OR "video game-based exercise"[Title/Abstract] OR "exercise game*"[Title/Abstract]) AND ("Ambulatory Care"[MeSH Terms] OR "Outpatients"[MeSH Terms] OR "outpatient*"[Title/Abstract] OR "Ambulatory Care"[Title/Abstract] OR "home-based rehabilitation"[Title/Abstract] OR "community-based rehabilitation"[Title/Abstract] OR "Physical Therapy Modalities"[MeSH Terms] OR "Exercise Therapy"[MeSH Terms] OR "physical rehabilitation"[Title/Abstract] OR "functional rehabilitation"[Title/Abstract] OR "motor rehabilitation"[Title/Abstract] OR "physical therapy"[Title/Abstract] OR "physiotherapy"[Title/Abstract])
**Scopus**	(TITLE-ABS-KEY ("stroke" OR "post-stroke" OR "ischemic stroke" OR "hemorrhagic stroke" OR "cerebrovascular accident" OR "brain infarction" OR "CVA" OR "transient ischemic attack" OR "brain ischemia" OR "intracranial hemorrhage" OR "cryptogenic stroke")) AND (TITLE-ABS-KEY ("virtual reality" OR "VR" OR "virtual environment*" OR "virtual rehabilitation" OR "exergam*" OR "active video game*" OR "interactive game*" OR "serious game*" OR "video game-based exercise" OR "exercise game*")) AND (TITLE-ABS-KEY ("outpatient*" OR "ambulatory care" OR "home-based rehabilitation" OR "community-based rehabilitation" OR "physical rehabilitation" OR "functional rehabilitation" OR "motor rehabilitation"))
**Embase**	('stroke'/exp OR 'brain ischemia'/exp OR 'cerebral hemorrhage'/exp OR 'intracranial thrombosis'/exp OR stroke:ti,ab OR 'post-stroke':ti,ab OR 'ischemic stroke':ti,ab OR 'hemorrhagic stroke':ti,ab OR 'cerebrovascular accident':ti,ab OR 'brain infarction':ti,ab OR cva:ti,ab OR 'transient ischemic attack':ti,ab OR 'brain ischemia':ti,ab OR 'intracranial hemorrhage':ti,ab OR 'cryptogenic stroke':ti,ab) AND ('virtual reality'/exp OR 'exergaming'/exp OR 'virtual reality':ti,ab OR vr:ti,ab OR 'virtual environment*':ti,ab OR 'virtual rehabilitation':ti,ab OR exergam*:ti,ab OR 'active video game*':ti,ab OR 'interactive game*':ti,ab OR 'serious game*':ti,ab OR 'video game-based exercise':ti,ab OR 'exercise game*':ti,ab) AND ('ambulatory care'/exp OR 'outpatient care'/exp OR outpatient*:ti,ab OR 'ambulatory care':ti,ab OR 'home-based rehabilitation':ti,ab OR 'community-based rehabilitation':ti,ab OR 'exercise therapy'/exp OR 'physical rehabilitation':ti,ab OR 'functional rehabilitation':ti,ab OR 'motor rehabilitation':ti,ab)
**Cochrane Trials**	#1 (stroke OR post-stroke OR ischemic stroke OR hemorrhagic stroke OR cerebrovascular accident OR brain infarction OR CVA OR transient ischemic attack OR brain ischemia OR intracranial hemorrhage OR cryptogenic stroke):ti,ab,kw<br>#2 (virtual reality OR VR OR virtual environment* OR virtual rehabilitation OR exergam* OR active video game* OR interactive game* OR serious game* OR video game-based exercise OR exercise game*):ti,ab,kw

	#3 (outpatient* OR ambulatory care OR home-based rehabilitation OR community-based rehabilitation):ti,ab,kw<br>#4 (physical rehabilitation OR functional rehabilitation OR motor rehabilitation OR physical therapy OR physiotherapy):ti,ab,kw<br>#5#1 AND #2 AND #3 AND #4
**Web of Science**	TS=("stroke" OR "post-stroke" OR "ischemic stroke" OR "hemorrhagic stroke" OR "cerebrovascular accident" OR "brain infarction" OR "CVA" OR "transient ischemic attack" OR "brain ischemia" OR "intracranial hemorrhage" OR "cryptogenic stroke") AND TS=("virtual reality" OR "VR" OR "virtual environment*" OR "virtual rehabilitation" OR "exergam*" OR "active video game*" OR "interactive game*" OR "serious game*" OR "video game-based exercise" OR "exercise game*") AND TS=("outpatient*" OR "ambulatory care" OR "home-based rehabilitation" OR "community-based rehabilitation" OR "physical rehabilitation" OR "functional rehabilitation" OR "motor rehabilitation")
