# Supplementary material for: Virtual Reality Exergaming in Outpatient Stroke Rehabilitation: A Scoping Review and Clinician Roadmap
Source: J Clin Med. 2025 Oct 13;14(20):7227. doi: 10.3390/jcm14207227 (PMC12565396; doi:10.3390/jcm14207227)
Supplement: Supplementary file 1 [file jcm-14-07227-s001.zip › Supplementary File S3.pdf]

Supplementary File S3. List of excluded studies with reason

Incorrect population:

1. Sin, H., & Lee, G. (2013). Additional virtual reality training using Xbox Kinect in stroke survivors with hemiplegia. *American journal of physical medicine & rehabilitation*, *92*(10), 871–880. https://doi.org/10.1097/PHM.0b013e3182a38e40
2. Yaman F, Akdeniz Leblebicier M, Okur İ, İmal Kızılkaya M, Kavuncu V. Is virtual reality training superior to conventional treatment in improving lower extremity motor function in chronic hemiplegic patients? Turk J Phys Med Rehabil. 2022 Aug 25;68(3):391-398. doi: 10.5606/tftrd.2022.9081.
3. Lee, M. M., Lee, K. J., & Song, C. H. (2018). Game-Based Virtual Reality Canoe Paddling Training to Improve Postural Balance and Upper Extremity Function: A Preliminary Randomized Controlled Study of 30 Patients with Subacute Stroke. *Medical science monitor : international medical journal of experimental and clinical research*, *24*, 2590–2598. https://doi.org/10.12659/MSM.906451
4. Kizony, R., Weiss, P. L. (T.), Shahar, M., & Rand, D. (2006). TheraGame: A home based virtual reality rehabilitation system. *International Journal on Disability and Human Development*, *5*(3), 265–269. https://doi.org/10.1515/IJDHD.2006.5.3.265
5. Chen, J., Or, C. K., Li, Z., Yeung, E. H. K., Zhou, Y., & Hao, T. (2023). Effectiveness, safety and patients' perceptions of an immersive virtual reality-based exercise system for poststroke upper limb motor rehabilitation: A proof-of-concept and feasibility randomized controlled trial. *Digital health*, *9*, 20552076231203599. https://doi.org/10.1177/20552076231203599
6. Saposnik, G., Cohen, L. G., Mamdani, M., Pooyania, S., Ploughman, M., Cheung, D., Shaw, J., Hall, J., Nord, P., Dukelow, S., Nilanont, Y., De Los Rios, F., Olmos, L., Levin, M., Teasell, R., Cohen, A., Thorpe, K., Laupacis, A., Bayley, M., & Stroke Outcomes Research Canada (2016). Efficacy and safety of non-immersive virtual reality exercising in stroke rehabilitation (EVREST): a randomised, multicentre, single-blind, controlled trial. *The Lancet. Neurology*, *15*(10), 1019–1027. https://doi.org/10.1016/S1474-4422(16)30121-1
7. Malik, A. N. & Masood, . T. (2017) Virtual reality training improves turning capacity and functional reach in stroke patients. Rawal Medical Journal, 42 (2), 158-161.
8. Park, M., Ko, M. H., Oh, S. W., Lee, J. Y., Ham, Y., Yi, H., Choi, Y., Ha, D., & Shin, J. H. (2019). Effects of virtual reality-based planar motion exercises on upper extremity function, range of motion, and health-related quality of life: a multicenter, single-blinded, randomized, controlled pilot study. *Journal of neuroengineering and rehabilitation*, *16*(1), 122. https://doi.org/10.1186/s12984-019-0595-8
9. Karasu, A. U., Batur, E. B., & Karataş, G. K. (2018). Effectiveness of Wii-based rehabilitation in stroke: A randomized controlled study. *Journal of rehabilitation medicine*, *50*(5), 406–412. https://doi.org/10.2340/16501977-2331
10. Lee, S., Kim, Y., & Lee, B. H. (2016). Effect of Virtual Reality-based Bilateral Upper Extremity Training on Upper Extremity Function after Stroke: A Randomized Controlled Clinical Trial. *Occupational therapy international*, *23*(4), 357–368. https://doi.org/10.1002/oti.1437
11. Aşkın, A., Atar, E., Koçyiğit, H., & Tosun, A. (2018). Effects of Kinect-based virtual reality game training on upper extremity motor recovery in chronic stroke. *Somatosensory & motor research*, *35*(1), 25–32. https://doi.org/10.1080/08990220.2018.1444599
12. M. Gorši ̌, I. Cikajlo, N. Goljar and D. Novak, "A Multisession Evaluation of a Collaborative Virtual Environment for Arm Rehabilitation," in *Presence*, vol. 27, no. 3, pp. 274-286, July 2020, doi: 10.1162/pres_a_00331.
13. Şimşek, T. T., & Çekok, K. (2016). The effects of Nintendo Wii(TM)-based balance and upper extremity training on activities of daily living and quality of life in patients with sub-acute stroke: a randomized controlled study. *The International journal of neuroscience*, *126*(12), 1061–1070. https://doi.org/10.3109/00207454.2015.1115993

14. Ozen, S., Senlikci, H. B., Guzel, S., & Yemisci, O. U. (2021). Computer Game Assisted Task Specific Exercises in the Treatment of Motor and Cognitive Function and Quality of Life in Stroke: A Randomized Control Study. *Journal of stroke and cerebrovascular diseases : the official journal of National Stroke Association*, *30*(9), 105991. https://doi.org/10.1016/j.jstrokecerebrovasdis.2021.105991
15. Grigoras A.V., Matei D., Ignat E.B. (2018). Non-Immersive Virtual Reality for Upper Limb Rehabilitation in Stroke Survivors- A Feasibility Study. Balneo Research Journal Balneo Research Journal, 9(3):232-239. 10.12680/balneo.2018.187
16. Iosa, M., Morone, G., Fusco, A., Castagnoli, M., Fusco, F. R., Pratesi, L., & Paolucci, S. (2015). Leap motion controlled videogame-based therapy for rehabilitation of elderly patients with subacute stroke: a feasibility pilot study. *Topics in stroke rehabilitation*, *22*(4), 306–316. https://doi.org/10.1179/1074935714Z.0000000036
17. Huang, Q., Jiang, X., Jin, Y., Wu, B., Vigotsky, A. D., Fan, L., Gu, P., Tu, W., Huang, L., & Jiang, S. (2024). Immersive virtual reality-based rehabilitation for subacute stroke: a randomized controlled trial. Journal of neurology, 271(3), 1256–1266. https://doi.org/10.1007/s00415-023-12060-y
18. Cho, H., Song, E., Moon, J-H., Hahm, S-C. (2021). Effects of Virtual Reality Based Therapeutic Exercise on the Upper Extremity Function and Activities of Daily Living in Patients with Acute Stroke: A Pilot Randomized Controlled Trial. *Medico Legal Update*, *21*(2), 676-682. https://doi.org/10.37506/mlu.v21i2.2761
19. Keskin, Y., Gürcan, A., Ürkmez, B., Akgül, Y.S.,Özaras, N., Aydin, T. (2020). Efficacy Of A Video-Based Physical Therapy And Rehabilitation System In Patients With Post-Stroke Hemiplegia: A Randomized, Controlled, Pilot Study. Turkish Journal Of Geriatrics, 23(1): 118-128. Doi: 10.31086/Tjgeri.2020.145
20. In, T., Lee, K., & Song, C. (2016). Virtual Reality Reflection Therapy Improves Balance and Gait in Patients with Chronic Stroke: Randomized Controlled Trials. *Medical science monitor : international medical journal of experimental and clinical research*, *22*, 4046–4053. https://doi.org/10.12659/msm.898157
21. Rodríguez-Hernández, M., Polonio-López, B., Corregidor-Sánchez, A. I., Martín-Conty, J. L., Mohedano-Moriano, A., & Criado-Álvarez, J. J. (2023). Can specific virtual reality combined with conventional rehabilitation improve poststroke hand motor function? A randomized clinical trial. *Journal of neuroengineering and rehabilitation*, *20*(1), 38. https://doi.org/10.1186/s12984-023-01170-3
22. Bao, X., Mao, Y., Lin, Q., Qiu, Y., Chen, S., Li, L., Cates, R. S., Zhou, S., & Huang, D. (2013). Mechanism of Kinect-based virtual reality training for motor functional recovery of upper limbs after subacute stroke. *Neural regeneration research*, *8*(31), 2904–2913. https://doi.org/10.3969/j.issn.1673-5374.2013.31.003
23. Miclaus, R., Roman, N., Caloian, S., Mitoiu, B., Suciu, O., Onofrei, R. R., Pavel, E., & Neculau, A. (2020). Non-Immersive Virtual Reality for Post-Stroke Upper Extremity Rehabilitation: A Small Cohort Randomized Trial. *Brain sciences*, *10*(9), 655. https://doi.org/10.3390/brainsci10090655
24. Ali, A. S., Kumaran, D. S., Unni, A., Sardesai, S., Prabhu, V., Nirmal, P., Pai, A. R., Guddattu, V., & Arumugam, A. (2024). Effectiveness of an Intensive, Functional, and Gamified Rehabilitation Program on Upper Limb Function in People With Stroke (EnteRtain): A Multicenter Randomized Clinical Trial. *Neurorehabilitation and neural repair*, *38*(4), 243–256. https://doi.org/10.1177/15459683231222921
25. Sheehy, L., Taillon-Hobson, A., Sveistrup, H., Bilodeau, M., Yang, C., & Finestone, H. (2020). Sitting Balance Exercise Performed Using Virtual Reality Training on a Stroke Rehabilitation Inpatient Service: A Randomized Controlled Study. *PM & R : the journal of injury, function, and rehabilitation*, *12*(8), 754–765. https://doi.org/10.1002/pmrj.12331

26. Lee, S. H., Lee, J. Y., Kim, M. Y., Jeon, Y. J., Kim, S., & Shin, J. H. (2018). Virtual Reality Rehabilitation With Functional Electrical Stimulation Improves Upper Extremity Function in Patients With Chronic Stroke: A Pilot Randomized Controlled Study. *Archives of physical medicine and rehabilitation*, *99*(8), 1447–1453.e1. https://doi.org/10.1016/j.apmr.2018.01.030
27. Rodríguez-Hernández, M., Polonio-López, B., Corregidor-Sánchez, A. I., Martín-Conty, J. L., Mohedano-Moriano, A., & Criado-Álvarez, J. J. (2021). Effects of Specific Virtual Reality-Based Therapy for the Rehabilitation of the Upper Limb Motor Function Post-Ictus: Randomized Controlled Trial. *Brain sciences*, *11*(5), 555. https://doi.org/10.3390/brainsci11050555
28. Eng, K., Siekierka, E., Pyk, P., Chevrier, E., Hauser, Y., Cameirao, M., Holper, L., Hägni, K., Zimmerli, L., Duff, A., Schuster, C., Bassetti, C., Verschure, P., & Kiper, D. (2007). Interactive visuo-motor therapy system for stroke rehabilitation. *Medical & biological engineering & computing*, *45*(9), 901–907. https://doi.org/10.1007/s11517-007-0239-1
29. Park, D. S., Lee, D. G., Lee, K., & Lee, G. (2017). Effects of Virtual Reality Training using Xbox Kinect on Motor Function in Stroke Survivors: A Preliminary Study. *Journal of stroke and cerebrovascular diseases : the official journal of National Stroke Association*, *26*(10), 2313–2319. https://doi.org/10.1016/j.jstrokecerebrovasdis.2017.05.019
30. Widmer, M., Held, J. P. O., Wittmann, F., Valladares, B., Lambercy, O., Sturzenegger, C., Palla, A., Lutz, K., & Luft, A. R. (2022). Reward During Arm Training Improves Impairment and Activity After Stroke: A Randomized Controlled Trial. *Neurorehabilitation and neural repair*, *36*(2), 140–150. https://doi.org/10.1177/15459683211062898
31. Lee, D., Lee, M., Lee, K., & Song, C. (2014). Asymmetric training using virtual reality reflection equipment and the enhancement of upper limb function in stroke patients: a randomized controlled trial. *Journal of stroke and cerebrovascular diseases : the official journal of National Stroke Association*, *23*(6), 1319–1326. https://doi.org/10.1016/j.jstrokecerebrovasdis.2013.11.006
32. Park, Y. H., Lee, C. H., & Lee, B. H. (2013). Clinical usefulness of the virtual reality-based postural control training on the gait ability in patients with stroke. *Journal of exercise rehabilitation*, *9*(5), 489–494. https://doi.org/10.12965/jer.130066
33. da Silva Cameirão, M., Bermúdez I Badia, S., Duarte, E., & Verschure, P. F. (2011). Virtual reality based rehabilitation speeds up functional recovery of the upper extremities after stroke: a randomized controlled pilot study in the acute phase of stroke using the rehabilitation gaming system. *Restorative neurology and neuroscience*, *29*(5), 287–298. https://doi.org/10.3233/RNN-2011-0599

Incorrect concept:

1. Toh, S. F. M., Gonzalez, P. C., & Fong, K. N. K. (2023). Usability of a wearable device for home-based upper limb telerehabilitation in persons with stroke: A mixed-methods study. *Digital health*, *9*, 20552076231153737. https://doi.org/10.1177/20552076231153737
2. Wilson PH, Rogers JM, Vogel K, Steenbergen B, McGuckian TB, Duckworth J. Home-based (virtual) rehabilitation improves motor and cognitive function for stroke patients: a randomized controlled trial of the Elements (EDNA-22) system. J Neuroeng Rehabil. 2021 Nov 25;18(1):165. doi: 10.1186/s12984-021-00956-7. PMID: 34823545; PMCID: PMC8613521.
3. Saposnik G, Chow C-M, Gladstone D, et al. iPad Technology for Home Rehabilitation after Stroke (iHOME): A Proof-of-Concept Randomized Trial. *International Journal of Stroke*. 2014;9(7):956-962. doi:10.1111/ijs.12328
4. Cano-Mañas, M. J., Collado-Vázquez, S., Rodríguez Hernández, J., Muñoz Villena, A. J., & Cano-de-la-Cuerda, R. (2020). Effects of Video-Game Based Therapy on Balance, Postural Control, Functionality, and Quality of Life of Patients with Subacute Stroke: A Randomized Controlled Trial. *Journal of healthcare engineering*, *2020*, 5480315. https://doi.org/10.1155/2020/5480315
5. Kilbride, C., Butcher, T., Warland, A., Ryan, J., Scott, D. J. M., Cassidy, E., Athanasiou, D. A., Singla-Buxarrais, G., Baker, K., & Norris, M. (2024). Rehabilitation via HOMe-Based gaming

exercise for the Upper limb post Stroke (RHOMBUS): a qualitative analysis of participants' experience. BMJ open, 14(1), e075821. https://doi.org/10.1136/bmjopen-2023-075821
6. El-Kafy, E. M. A., Alshehri, M. A., El-Fiky, A. A., & Guermazi, M. A. (2021). The Effect of Virtual Reality-Based Therapy on Improving Upper Limb Functions in Individuals With Stroke: A Randomized Control Trial. *Frontiers in aging neuroscience*, *13*, 731343. https://doi.org/10.3389/fnagi.2021.731343

Incorrect context:

1. Baluz R, Teles A, Fontenele JE, et al. Motor Rehabilitation of Upper Limbs Using a Gesture-Based Serious Game: Evaluation of Usability and User Experience. *Games Health J.* 2022;11(3):177-185. doi:10.1089/g4h.2022.0005
2. Bouatrous, A., Meziane, A., Zenati, N. *et al.* A new adaptive VR-based exergame for hand rehabilitation after stroke. *Multimedia Systems* **29**, 3385–3402 (2023). https://doi.org/10.1007/s00530-023-01180-0
3. C. -H. Shih, P. -J. Lin, Y. -L. Chen and S. -L. Chen, "A Post-Stroke Rehabilitation System With Compensatory Movement Detection Using Virtual Reality and Electroencephalogram Technologies," in *IEEE Access*, vol. 12, pp. 61418-61432, 2024, doi: 10.1109/ACCESS.2024.3392513.
4. Navea, RF, Talde, VM, Armintia, FL, Cruz, SMD, Medina, G & Decena, A 2025, 'Gamified shoulder rehabilitation for mild stroke patients using virtual reality', *International Journal of Technology*, vol. 16, no. 1, pp. 146-159
5. Villada Castillo, J. F., Montoya Vega, M. F., Muñoz Cardona, J. E., Lopez, D., Quiñones, L., Henao Gallo, O. A., & Lopez, J. F. (2024). Design of Virtual Reality Exergames for Upper Limb Stroke Rehabilitation Following Iterative Design Methods: Usability Study. *JMIR serious games*, *12*, e48900. https://doi.org/10.2196/48900
6. Neil, A., Ens, S., Pelletier, R., Jarus, T., & Rand, D. (2013). Sony PlayStation EyeToy elicits higher levels of movement than the Nintendo Wii: implications for stroke rehabilitation. *European journal of physical and rehabilitation medicine*, *49*(1), 13–21.
7. Standen, P. J., Threapleton, K., Connell, L., Richardson, A., Brown, D. J., Battersby, S., Sutton, C. J., & Platts, F. (2015). Patients' use of a home-based virtual reality system to provide rehabilitation of the upper limb following stroke. *Physical therapy*, *95*(3), 350–359. https://doi.org/10.2522/ptj.20130564
8. C. Gmez-Portes, D. Carneros-Prado, J. Albusac, J. J. Castro-Schez, C. Glez-Morcillo and D. Vallejo, "PhyRe Up! A System Based on Mixed Reality and Gamification to Provide Home Rehabilitation for Stroke Patients," in *IEEE Access*, vol. 9, pp. 139122-139137, 2021, doi: 10.1109/ACCESS.2021.3118842.
9. Juan, M. C., Elexpuru, J., Dias, P., Santos, B. S., & Amorim, P. (2023). Immersive virtual reality for upper limb rehabilitation: comparing hand and controller interaction. *Virtual reality*, *27*(2), 1157–1171. https://doi.org/10.1007/s10055-022-00722-7
10. Song, X., van de Ven, S. S., Chen, S., Kang, P., Gao, Q., Jia, J., & Shull, P. B. (2022). Proposal of a Wearable Multimodal Sensing-Based Serious Games Approach for Hand Movement Training After Stroke. *Frontiers in physiology*, *13*, 811950. https://doi.org/10.3389/fphys.2022.811950
11. Ballester, B. R., Nirme, J., Duarte, E., Cuxart, A., Rodriguez, S., Verschure, P., & Duff, A. (2015). The visual amplification of goal-oriented movements counteracts acquired non-use in hemiparetic stroke patients. *Journal of neuroengineering and rehabilitation*, *12*, 50. https://doi.org/10.1186/s12984-015-0039-z
12. Bedendo, V., Aruanno, B., Dozio, N., Covarrubias, M., Ferrise, F., Bordegon, M. (2024). Exploiting Virtual Reality to Design Exercises for the Recovery of Stroke Patients at Home Computer-Aided Design and Applications, 21(3), 463-473. https://doi.org/10.14733/cadaps.2024.463-473
13. Baranyi, R., Körber, Y., Galimov, P., Parandeh, Z., Grechenig. T. (2023). Rehafox – A therapeutical approach developing a serious game to support rehabilitation of stroke patients

using a leap motion controller, Clinical eHealth, 6, 85-95. https://doi.org/10.1016/j.ceh.2023.08.001
14. Triandafilou, K. M., Tsoupikova, D., Barry, A. J., Thielbar, K. N., Stoykov, N., & Kamper, D. G. (2018). Development of a 3D, networked multi-user virtual reality environment for home therapy after stroke. *Journal of neuroengineering and rehabilitation*, *15*(1), 88. https://doi.org/10.1186/s12984-018-0429-0
15. Rand, D., Givon, N., Weingarden, H., Nota, A., & Zeilig, G. (2014). Eliciting upper extremity purposeful movements using video games: a comparison with traditional therapy for stroke rehabilitation. *Neurorehabilitation and neural repair*, *28*(8), 733–739. https://doi.org/10.1177/1545968314521008
