# Supplementary material for: Virtual Reality Exergaming in Outpatient Stroke Rehabilitation: A Scoping Review and Clinician Roadmap
Source: J Clin Med. 2025 Oct 13;14(20):7227. doi: 10.3390/jcm14207227 (PMC12565396; doi:10.3390/jcm14207227)
Supplement: Supplementary file 1 [file jcm-14-07227-s001.zip › Supplementary File S4.pdf]

Supplementary Files S4

Table S1. Characteristics of VR exergaming in clinic-based setting.

Study	VR system/platform	Interaction modality	Targeted Domain	Key outcomes and findings
**Serious games**				
Jo et al. (2024)[1]	360° VR mirror therapy (Pico G2 4K HMD)	None (visual mirror feedback via HMD)	Upper Limb	VR mirror therapy group showed significantly greater improvement in upper extremity motor function (FMA-UE, MFT, BBT) and higher satisfaction than traditional mirror therapy or conventional therapy.
Lesmana et al., 2024[2]	Custom VR cycling system (sensorized recumbent bike + screen VR)	Recumbent cycle with pedal force sensors & HR monitor	Lower Limb	After 12 weeks of VR cycling, stroke participants showed significant within-group improvements in mobility and endurance: faster Timed Up & Go, increased 6-Minute Walk distance, and higher VO_2max.
Peláez-Vélez et al. (2023)[3]	Oculus Quest 2 HMD + Kinect sensor (Blexer platform game "Phiby's Adventure")	Head-mounted display + full-body tracking via depth sensor	Multiple	Immersive VR exercise (mini-games for chopping, rowing, climbing) produced greater improvements than conventional therapy in motor function (Motricity Index), trunk control (TCT), and balance (Tinetti score, BBS). Strength and spasticity outcomes were similar between groups.
Hsu et al. (2022)[4]	Oculus Rift HMD + Leap Motion (VR mirror therapy software)	Hand tracking sensor (Leap Motion)	Upper Limb	No significant group difference on total FMA-UE after 9 weeks of VR mirror therapy vs controls. However, the VR mirror group showed greater improvement in wrist movement (FMA wrist subscore) compared to conventional therapy, and better hand dexterity than traditional mirror therapy.
Junata et al. (2021)[5]	Kinect-based Rapid Movement Training platform	Depth camera	Balance	Within-group improvements on BBS, TUG, and FMA matched conventional balance training. Faster rectus femoris activation during lean-and-release, with increased step displacement.
Escalante-Gonzalbo et al. (2021)[6]	LANR platform (custom serious games) with Kinect, Leap Motion, glove	Depth camera + hand tracker + standard controllers	Upper Limb	After 20 weeks of home-supported play, the Wolf Motor Function Test score improved significantly (+5.44 points, p=0.0039). Arm function (CAHAI) did not change significantly; the program was reported safe and well accepted by users.
Xu et al. (2021)[7]	Custom "Stomp Joy" game with depth camera and display	Depth camera (full-body motion capture)	Lower Limb	The Stomp Joy VR group showed significantly longer single-leg stance time and positive but not statistically confirmed for FMA-LE/MBI/BBS compared to control.
Johnson et al. (2020)[8]*	Jintronix rehabilitation system (Kinect-based exergames)	Depth camera (Microsoft Kinect)	Upper Limb	Virtual therapy led to significantly greater improvement in FMA-UE than usual care (p=0.02, moderate effect). Spasticity decreased more in VR, and MAL arm-use improved in VR (QOM better than control); ARAT and EQ-5D showed no group difference; high adherence and no adverse events.
Lee et al. (2020) [9]*	RAPAEL Smart Glove system (hand motion glove + rehab games)	Sensor glove (motion and orientation tracking)	Upper Limb	Glove-based VR therapy produced greater gains than control in hand dexterity (Box & Blocks, p<0.001), grip strength (p=0.001), and arm function (WMFT, p=0.032). No significant differences in Jebsen-Taylor Hand Function or Trail Making (cognitive) tests.

Norouzi-Gheidari et al. (2020)[10]*	Jintronix system (custom upper-limb rehab games with Kinect)	Depth camera (Kinect)	Upper Limb	After 4 weeks, the VR + standard rehab group showed higher Motor Activity Log quality-of-movement scores and better Stroke Impact Scale (mobility and physical domains) than control. No serious adverse events; clinic delivery was feasible.
Ahmad et al. (2019)[11]	Custom PC-based games with CyWee Z motion controller (dual-handle bar input)	Motion controller (accelerometer/gyroscope-based)	Upper Limb	Both the VR exergaming group and the standard physiotherapy group showed significant pre-to-post improvements in upper-limb function (FMA-UE, WMFT) and some ADL measures. There were no significant differences between groups; in fact, the control group had greater gains in IADL independence and intrinsic motivation than the VR group.
Henrique et al. (2019)[12]	Motion Rehab AVE 3D (Kinect-based system with projector display)	Depth camera (Kinect)	Multiple	VR group had significantly greater FMA-UE improvements (especially shoulder/elbow component, p=0.001) than conventional therapy. Both groups improved balance (BBS) similarly (between-group p=0.054).
Hung et al. (2019)[13]	Kinect2Scratch platform (custom video-capture games on PC)	Depth camera (Kinect)	Upper Limb	No significant between-group differences were found in any clinical outcome post-intervention or at 3-month follow-up. Both the Kinect2Scratch group and the therapist-trained group improved similarly in FMA-UE, WMFT, and MAL (all $p > 0.05$). Both groups achieved comparable gains, although the VR group demonstrated higher active use of the affected arm.
Lupo et al. (2018)[14]*	RIABLO™ rehab system (wireless IMUs on body + force platform games)	IMU motion sensors (trunk/legs) + Balance force plate	Balance	The VR (RIABLO) group showed significantly greater improvement in balance (BBS, p=0.008) and neurologic impairment (NIHSS, p=0.03) than conventional therapy. Center-of-pressure sway decreased over time in both eyes-open and closed conditions; patient participation and engagement were higher with VR, and no adverse events occurred.
Noveletto et a. (2018)[15]	Custom balance board system (IMU sensor board + myBalance software on PC)	Instrumented balance board (inertial sensors, real-time visual feedback)	Balance	After 10 weeks of balance exergaming, significant improvements were observed: BBS increased by ~5.3 points (ES=0.9, p<0.001), TUG time decreased ~3.6 s (p<0.001), and center-of-pressure sway reduced (121→76 cm path length, p<0.001). Game performance scores (balance tasks) improved markedly (large effect sizes).
Perez-Marcos et al. (2017)[16]*	MindMotion PRO system (3D motion camera + wrist IMUs, avatar feedback)	Depth camera + wrist IMU sensors	Upper Limb	High-intensity outpatient VR training was feasible with no serious adverse events. FMA-UE scores improved (~15% median increase) by 1-month follow-up, with significant gains in shoulder and forearm range of motion; no change in FIM independence.
Stockley et al. (2017)[17]	YouGrabber system (sensor gloves with custom rehab games)	Sensor gloves (hand motion tracking)	Upper Limb	There were no significant between-group differences between the YouGrabber and conventional gym training after 12 weeks. However, significant within-group improvements were observed in the VR group: the MAL scores improved in the YouGrabber group (median MAL increase +0.59 on amount-of-use, $p < 0.05$), whereas the control group's MAL change was smaller and not significant.
Lloréns et al. (2015)[18]	Custom VR stepping exercise (screen-based balance games)	Stepping movements tracked via sensors (pressure pad/Kinect)	Balance	The VR stepping group achieved greater balance gains on the Berg Balance Scale and faster 10m walking than conventional therapy. More VR participants progressed to a higher functional balance level; no significant side effects reported.

Tsoupikova et al. (2015)[19]	Custom semi-immersive VR (dual 3D displays, PneuGlove with force-feedback, IMU trackers)	Instrumented glove (finger bend sensors + pneumatic assistance) + motion trackers	Upper Limb	Participants improved their virtual task performance time by ~38% (p<0.05) over 6 weeks. Clinically, only lateral pinch strength improved significantly post-training (p=0.028) but was not maintained at 1 month; other grip and arm function measures showed no significant group changes.
Crosbie et al. (2012)[20]	Custom immersive VR (HMD + electromagnetic trackers for arm/hand)	Electromagnetic motion sensors (arm/hand tracking)	Upper Limb	No significant differences between VR and conventional therapy in Motricity Index or ARAT after 3 weeks. Both groups had small, non-clinically significant gains in arm function; VR had high adherence, with 1 dropout and only minor transient dizziness reported.
Levin et al. (2012)[21]*	GestureTek IREX video capture VR system (user on-screen in virtual tasks)	Video camera motion capture (no wearable input)	Upper Limb	Both VR and conventional OT groups improved in impairment and function, but more VR participants achieved clinically significant gains on FMA-UE and WMFT earlier. No differences in MAL (activity use); VR was well accepted with modest overall advantage.
Kim et al. (2009)[22]*	GestureTek IREX video capture VR system (user on-screen in virtual tasks)	Video capture camera (full-body tracking)	Multiple	Combined VR + PT yielded greater improvements in balance (BBS) and dynamic stability (postural sway angles) than PT alone (ANOVA p<0.05). VR participants also improved gait parameters (cadence, step length) more, and improvements in gait correlated with balance gains.
Broeren et al. (2008)[23]	Workbench VR with Phantom Omni haptic stylus + shutter glasses (3D games)	Haptic stylus (force-feedback pen)	Upper Limb	VR training led to ~9% improvement in Box & Blocks Test vs no change in controls. No clinically relevant change in hand function (ABILHAND), but VR group improved movement kinematics more than controls.
Yang et al. (2008)[24]	CAVE-style VR treadmill (triple-screen ~154° display + leg sensors)	Treadmill walking + Electromagnetic leg trackers	Gait	VR treadmill training improved gait speed over 10m and 400m more than treadmill without VR. At follow-up, community mobility (WAQ) was better in VR group; balance confidence improved only in VR group.
Commercial Exergame				
Korkusuz et al. (2025)[25]	Xbox 360 Kinect skiing games (Skicross, Bounty Slope) with visual biofeedback	Depth camera (Kinect)	Lower Limb	Both VR+PT and control PT improved balance and knee control in chronic stroke patients with knee hyperextension gait. The VR group showed greater improvements in Functional Reach and TUG (faster times) at post-test, while knee hyperextension angle and BI improved similarly in both groups.
Farahmand et al. (2024)[26]	Nintendo Wii + Balance Board	Balance Board (force plate) + Wii Remote	Lower Limb	Within the VR group, ankle spasticity scores (Modified Ashworth) improved significantly (p=0.001). However, there were no significant between-group differences in spasticity reduction or H-reflex measures compared to conventional therapy.
Sultan et al., 2023[27]	**Xbox Kinect** exergaming (Kinect Sports/Adventures)	Depth camera (Kinect)	Balance	Between-group improvements favored Kinect for TUG, TIS, and FIM; BBS improved in both groups with no between-group difference (p = 0.08).
Ali et al. (2022)[28]	Nintendo Wii Fit (Balance Board games) with added	Balance Board + Wii Remote	Balance	VR balance training with cognitive dual-tasks led to significantly better balance scores (BBS) and reduced fall risk (FES-I) compared to conventional balance training. Between-group differences were significant (BBS p=0.001), favoring the VR dual-task group.

	dual-task cognitive exercises			
Anwar et al. (2022)[29]	Nintendo Wii (Wii Sports boxing/tennis, Cooking Mama, Wii Fit board)	Motion controllers (Wii Remote) + Balance Board	Multiple	After 6 weeks, the VR group showed significantly better balance (BBS, $p<0.001$) and upper extremity motor scores (FMA-UE, $p=0.03$) than routine PT. VR also led to greater improvements in joint range of motion and reduced pain ($p<0.001$).
Ul et al. (2022)[30]	Xbox 360 Kinect (Kinect Sports/Adventures games for upper limb)	Depth camera (Kinect)	Upper Limb	Wii/Kinect exergaming (35 min, 5×/week) led to significantly better arm function (WMFT performance, $p<0.001$) in the VR group compared to conventional PT. Both groups improved spasticity (MAS) similarly, with no between-group difference.
Anwar et al. (2021)[31]	Nintendo Wii (Wii Fit balance games, Wii Sports aerobics) in clinic	Balance Board + Wii Remote + body-weight support (parallel bars)	Multiple	VR balance training led to significantly greater improvements in balance (BBS) and lower-limb motor recovery (FMA-LE motor) compared to dose-matched PT. The VR group also had better improvements in lower limb range of motion and pain reduction; sensory outcomes did not differ.
Ain et al. (2021) [32]	Xbox 360 Kinect (exergaming with Tennis, Joy Riding, Rally Ball)	Depth camera (Kinect motion tracking)	Upper Limb	Kinect group had significantly greater FMA-UE gains than conventional therapy (total and sub-scores). No cognitive benefit (MOCA), and both groups improved similarly in Box & Block dexterity.
Marques-Sule et al., 2021[33]	Nintendo Wii (Wii Fit balance board + Wii Sports)	Nintendo Wii (Wii Fit balance board + Wii Sports)	Multiple	Significant group×time effects favoring VR+PT on TUG, POMA, and BBS; post-hoc between-group differences favored VR; ADLs (Barthel, FAI) improved within VR group only; upper-limb impairment (FMA-UL) showed no between-group change.
Malik et al. (2021) [34]	Xbox 360 Kinect (Kinect Sports/Kinect Adventures games for balance)	Depth camera (Kinect)	Multiple	VR balance training resulted in significantly greater improvements than task-oriented training in lower-extremity motor scores (FMA-LE), balance (Berg Balance), and mobility (TUG) by week 8. Both groups improved gait (DGI) similarly.
Kannan et al. (2019)[35]	Nintendo Wii Fit with simultaneous cognitive tasks	Balance Board + Wii Remote	Balance	Combined balance and cognitive training with Wii Fit significantly improved volitional balance control ($p=0.001$) compared to control. Both dual-task VR and single-task training improved reactive balance and clinical measures (BBS, TUG, 6MWT) similarly; the VR group demonstrated better cognitive task accuracy during balance tasks.
Miranda et al. (2019)[36]	Nintendo Wii Fit (Balance Board games: Table Tilt, Tilt City, etc.)	Balance Board	Balance	Game performance improved across sessions, but there were no significant improvements in standardized balance outcomes (Limits of Stability or Rhythmic Weight Shift on Balance Master) in either the VR or control group.
Subramaniam & Bhatt (2019)[37]	Microsoft Kinect "Just Dance 3" game	Depth camera (Kinect body tracking)	Multiple	This preliminary study found that an intensive 6-week dance exergaming regimen was feasible and safe. Participants demonstrated faster movement initiation and improved shoulder movement times (from EMG/kinematic analysis), as well as trends toward better balance confidence (ABC) and mobility (TUG), although detailed statistical outcomes were not reported in the summary.
Carregosa et al. (2018)[38]	Nintendo Wii (Wii Sports games with handheld controller)	Motion controller (Wii Remote)	Multiple	In this small pilot, 16 sessions of Wii-based training led to descriptive improvements in Fugl-Meyer upper-limb motor scores and total motor scores, which were maintained at 2-

				month follow-up. Participants also showed improvements in SF-36 functional capacity, though statistical tests were limited by the small sample.
Pedreira da Fonseca et al. (2017)[39]	Nintendo Wii (Wii Sports games: Tennis, Hula Hoop, Soccer, Boxing)	Motion controllers (Wii Remote) + Balance Board	Balance	Both Wii exergaming and conventional physiotherapy groups improved over 2 months. The control group had a significant improvement in gait balance (DGI), and the VR group saw a reduction in falls count (pre vs post, p=0.049). No significant between-group differences in DGI or falls were found.
da Silva Ribeiro et al. (2015)[40]	Nintendo Wii (Wii Sports games via projector display)	Motion controllers (Wii Remote + Nunchuk)	Multiple	Both the Wii group and conventional PT group showed significant improvements in Fugl-Meyer motor scores (upper limb, lower limb, balance) and in multiple SF-36 quality-of-life domains. There were no significant between-group differences in motor outcomes; only the SF-36 Physical Functioning domain favored the control group.
Hung et al. (2014)[41]	Nintendo Wii Fit (Balance Board exercises) vs weight-shifting training	Balance Board	Balance	Both the Wii Fit exergaming and conventional weight-shifting therapy produced significant improvements in dynamic balance (TUG, Forward Reach) and reduced fear of falling. The exergaming group enjoyed the training more and showed specific improvements in certain posturography stability measures during training, though these effects were not fully maintained at 3-month follow-up.
Fritz et al. (2013) [42]	Nintendo Wii & PlayStation EyeToy (Wii Sports, Wii Fit, EyeToy)	Motion controller (Wii Remote) + Camera (EyeToy)	Multiple	VR gaming group showed small positive effects on balance (BBS) and mobility (DGI, gait tests) vs control, with larger effect sizes but no statistically significant differences.
Singh et al. (2013)[43]	Nintendo Wii Fit Plus (Balance Board) + Xbox 360 Kinect (Rally Ball game)	Balance Board + Depth camera	Multiple	After 6 weeks of intervention (VR + PT vs extended PT), both groups showed improvements in functional mobility (TUG, 10m Walk, 6MWT) and independence (Barthel). The VR adjunct did not produce statistically significant additional benefits over standard therapy in this trial (outcomes were similar between groups), but the approach was considered feasible and safe.
Reinthal et al. (2012)[44]	ENGAGE protocol with Nintendo Wii & PS2 EyeToy games	Motion controller (Wii Remote) + Camera (EyeToy)	Upper Limb	Participants in the VR group engaged in high doses of practice (mean ~1089 min over study). Significant within-group gains were seen in FMA-UE (+8 points, p=0.002) and faster WMFT (−11 s, p=0.003); no serious adverse events, and motivation scores were higher for gaming than for traditional exercise.

10MWT: 10-Meter Walk Test; 3D: three-dimensional; 6MWT: 6-Minute Walk Test; ABC: Activities-specific Balance Confidence scale; ADL: activities of daily living; ANOVA: analysis of variance; ARAT: Action Research Arm Test; BBB: Box and Block Test; BBS: Berg Balance Scale; BI: Barthel Index; CAHAI: Chedoke Arm and Hand Activity Inventory; CAVE: Cave Automatic Virtual Environment; DGI: Dynamic Gait Index; EMG: electromyography; EQ-5D: EuroQol 5-Dimensions; FAI: Frenchay Activities Index; FES-I: Falls Efficacy Scale International; FIM: Functional Independence Measure; FMA-LE: Fugl-Meyer Assessment Lower Extremity; FMA-UE: Fugl-Meyer Assessment Upper Extremity; HMD: head-mounted display; H-reflex: Hoffmann reflex; HR: heart rate; IADL: instrumental activities of daily living; IMU: inertial measurement unit; MAL: Motor Activity Log; MAS: Modified Ashworth Scale; MBI: Modified Barthel Index; MFT: Manual Function Test; MoCA: Montreal Cognitive Assessment; NIHSS: National Institutes of Health Stroke Scale; OT: occupational therapy; POMA: Performance Oriented Mobility Assessment; PT: physical therapy; QOM: quality of movement; ROM: range of motion; SF-36: 36-Item Short Form Health Survey; SIS: Stroke Impact Scale; TCT: Trunk Control Test; TIS: Trunk Impairment Scale; TUG: Timed Up and Go; UE: upper extremity; LE: lower extremity; UL: upper limb; VO_2max: maximal oxygen uptake; WAQ: Walking Ability Questionnaire; WMFT: Wolf Motor Function Test; VR: virtual reality. *platform commercially available.

Table S2. Characteristics of VR exergaming in home-based setting.

Study	VR system/platform	Interaction modality	Targeted domain	Key outcomes and findings
**Serious games**				
Ase et al. (2025)[45]*	RAPAEL Smart Glove system (sensorized glove + tablet-based VR tasks)	Sensor glove (strain gauge, accelerometer, goniometer)	Upper Limb	Home-based glove training led to greater and sustained improvements than a conventional home exercise program on FMA-UE, Jebsen-Taylor hand function, Box & Block, and MAL (Amount of Use). The VR group exercised ~1.8× longer and had no adverse events.
Sheehy et al. (2025)[46]*	Jintronix Home (Kinect-based balance and mobility games via tele-rehab)	Depth camera (Kinect body tracking)	Multiple	In this feasibility RCT, both the home VR exergaming group and the active control (iPad apps) improved significantly over 6 weeks on balance (BBS), various TUG tests, and functional measures. There were no significant differences between groups; the program was deemed safe with no serious adverse events.
Fluet et al. (2024)[47]	Home-based VR Rehab (HoVRS) – Leap Motion controller with Unity 3D games	Optical hand tracking (Leap Motion)	Upper Limb	In this trial of two adaptive algorithms, there were no significant group differences; however, across both groups, participants improved upper-limb motor function (mean FMA-UE +5.85) and ARAT hand function, and reported stable or increased intrinsic motivation. Adherence was good and no adverse events were noted.
Lu et al. (2024)[48]	Kinect V2–based home guidance system	Depth camera (Kinect v2)	Multiple	Pre–post improvements were observed in joint range of motion and balance control with reductions in spasticity, whereas Berg Balance Scale change was not significant overall; total training time was ~800 minutes over ~2 months in a small single-group study.
Desai et al. (2023)[49]	Custom PIXER home system (Kinect V2 RGB-D sensor + Unity-based "bubble burst" exercises)	Depth camera (Kinect v2)	Upper Limb	A one-month personalized VR home exercise program was feasible: all participants who completed reported improved physical function on SIS (trend ↑). At 3 months, 77% of personal rehab goals were achieved; no adverse events occurred, though retention dropped to 50%. In-game metrics (repetitions, hit rate) improved over time, indicating increased performance.
Chen et al. (2022)[50]	Immersive home VR system (HMD + electromagnetic tracking; custom 3D reach-to-grasp games)	Electromagnetic motion tracking (hand/arm)	Upper Limb	After a 6-week immersive VR home program, participants demonstrated significant improvements in functional independence (FIM +5.5, $p<0.01$) and self-reported health status (EQ-5D VAS +7.1, $p<0.01$). Quality-of-life index scores also improved modestly.
Ellis et al. (2022)[51]*	EvolvRehab Body platform (laptop + Kinect v2 + Leap Motion; personalized exergames)	Depth camera + hand tracking sensor	Upper Limb	Eight chronic stroke survivors completed a 12-week home program. Some participants demonstrated clinically meaningful improvements in WMFT (time and functional scores); grip strength did not show notable changes, and ARAT and Motricity Index results varied across individuals. Remote

				surface EMG monitoring was feasible, although data quality was limited for the more-affected limb.
Kilbride et al. (2022)[52]*	Neurofenix NeuroBall and NeuroBands with tablet (7 upper-limb exercise games)	Hand and arm sensor units (inertial sensors in ball and bands)	Upper Limb	A 7-week home program with the NeuroBall/Bands was feasible. Significant within-group gains were observed in FMA-UE (+2.68, 95% CI 0.86–4.49) and a slight increase in arm use (MAL +0.19) by 12 weeks. Shoulder, elbow, and wrist passive range of motion improved, and user acceptability was high (QUEST questionnaire). There were 30 mild adverse events recorded (e.g. transient aches), but no serious issues.
Lansberg et al(2021)[53]*	Neofect/RAPAEL Smart Glove with tablet (ADL-themed hand therapy games)	Sensor glove (accelerometers, flex sensors) + tablet app	Upper Limb	Eight weeks of home glove gaming led to significant improvements in hand function: Jebsen-Taylor test times decreased by ~26.6 s (especially fine motor sub-tasks, p=0.03) and Stroke Impact Scale hand domain increased (+16.1 points, p<0.01). FMA-UE improved by +2.2 points on average (non-significant, p=0.10). No adverse events; user satisfaction was high (80–95% positive feedback).
Jonsdottir et al. (2021)[54]	HEAD rehab platform (Kinect + Leap Motion for motor-cognitive exergames)	Depth camera + optical hand tracker	Multiple	A hybrid clinic/home VR program (including dual-task exercises) showed at 3-month follow-up, the VR group maintained walking endurance (2-minute walk) significantly better than the usual care group. No serious adverse events occurred; adherence was high both in clinic (92%) and at home (89%), and usability was good (SUS ~77.5/100).
Thielbar et al. (2020)[55]	VERGE exergame (home laptop + Kinect; networked multi-user vs single-user games)	Depth camera (Kinect) + online multiplayer connectivity	Upper Limb	In a crossover comparison, multiplayer VR sessions induced longer active exercise time and greater arm movement distance than single-player sessions. Overall upper-limb motor function improved (FMA-UE +3.2 points, p=0.001) over 4 weeks of VR training; compliance was very high (99% of scheduled multiplayer sessions attended) and participants reported high engagement, with no serious adverse events.
Qiu et al. (2020)[56]	Home-based HoVRS (Leap Motion controller + PC, library of 12 therapy games)	Optical hand tracking (Leap Motion)	Upper Limb	Twelve weeks of Leap Motion home training yielded a mean FMA-UE improvement of +5.2 points (SE 0.69, p<0.001), exceeding the minimal clinically important difference. Hand range-of-motion increased (e.g. wrist pronation +27.5%) and movement accuracy errors decreased; no adverse events occurred. Combinations of kinematic metrics significantly predicted FMA gains, suggesting potential for remote monitoring.
Borstad et al. (2018)[57]	Recovery Rapids game (adaptive kayaking-themed VR) with Xbox 360 Kinect + instrumented glove	Depth camera (Kinect) + custom glove (flex sensors, accelerometers)	Upper Limb	An intensive 10-day program (3 hours/day) of VR gaming produced large improvements in arm function: the Wolf Motor Function Test speed improved substantially (effect size d=1.5, p<0.001) and MAL quality-of-movement increased (d=1.1, p<0.001). No serious adverse events occurred, though not all feasibility criteria were met (issues with glove use and MAL homework adherence).

Ballester et al. (2017)[58]	Rehabilitation Gaming System (RGS) – Kinect camera + data gloves ("Spheroids" game tasks)	Depth camera + sensor gloves	Upper Limb	After 3 weeks, the home RGS group had a significantly greater improvement in arm function (CAHAI, d=0.48, p≈0.05) than the control exercise group. No significant between-group difference in FMA-UE was seen at that point, and gains were not maintained by 12-week follow-up.
Standen et al. (2017)[59]	Virtual Glove home system (infrared glove + Wii remote controllers, custom games)	Sensor glove (infrared) + motion controllers	Upper Limb	VR home intervention users had greater gains in grip strength (Wolf test) and arm activity (Motor Activity Log) compared to controls. However, many patients required substantial support for system setup and use.
Nijenhuis et al. (2016)[60]	SCRIPT system (sensorized passive wrist-hand orthosis + SaeboMAS support, custom PC games)	Instrumented arm orthosis (sensors for wrist/hand motions)	Upper Limb	After 6 weeks, both the home VR (orthosis gaming) and control exercise groups showed moderate, similar improvements in arm function. No significant between-group differences were found on the primary outcome (ARAT) or other clinical measures, though longer training duration was correlated with better dexterity (Box & Blocks) outcomes in the VR group.
Wittmann et al. (2016)[61]*	ArmeoSenso home system (3 wearable IMUs for arm/trunk, therapy games on PC)	Inertial measurement units (upper arm, forearm, trunk)	Upper Limb	Home use of the ArmeoSenso was feasible and safe (no adverse events). Participants achieved significant arm motor gains (FMA-UE +4.1, p=0.003), while WMFT times were not significantly changed; sensor metrics (workspace area +31%, target time −19%) improved, and users maintained high motivation (training dose did not drop over 6 weeks).
Zondervan et al. (2016)[62]*	MusicGlove system (sensorized glove with finger pinch sensors + rhythm-game software)	Sensor glove (fingertip switches) + laptop	Upper Limb	After a 3-week home program (≥3 hours/week), there was no significant difference in Box & Blocks improvement between the MusicGlove group and tabletop exercise group at 1-month follow-up. However, the MusicGlove group showed greater gains in hand function use (MAL Quality of Movement +0.72, p=0.007; MAL Amount of Use +0.60, p=0.04). The intervention was feasible with increasing self-driven use over time and no adverse events.
Slijper et al. (2014)[63]	Custom home gaming system (3D hand-position tracking via two grip sensors on strings; 15-game suite)	Custom 3D hand tracking (handle grips on strings, positional sensors)	Upper Limb	Over 5 weeks of unsupervised home play (~1070 minutes total on average), FMA-UE synergy scores (A→D) and ARAT improved significantly from baseline to post-test and were maintained at follow-up. Grip strength on the affected side increased, although self-reported manual ability (ABILHAND) did not significantly change. No correlation was found between training dose and outcomes.
**Commercial Exergame**				
Golla et al., 2018[64]	Nintendo Wii Fit with Wii Balance Board	Force-plate weight-shift with visual feedback in Wii balance games	Balance	Home use was feasible and safe with no falls or injuries; baseline ceiling effects limited sensitivity, and no significant between-group differences were detected on balance outcomes over the 6-week home phase.

Adie et al. (2016)[65]	Nintendo Wii Sports games (bowling, tennis, golf, etc.) with Wii Remote at home	Motion controller (Wii Remote)	Upper Limb	No significant difference was found between the Wii exergaming group and the home exercise group in upper extremity function (ARAT) after 6 weeks. Both groups improved, and there were no serious adverse events; the Wii approach was more costly with a high probability of being less cost-effective.
Rand et al. (2016)[66]	Microsoft Xbox Kinect or Sony PlayStation 2 EyeToy (home-based)	Video-capture consoles: depth sensor/camera tracking; mostly standing for Kinect, seated for EyeToy; weaker-arm movements emphasized	Upper limb	No significant between-group differences vs GRASP. Within-group ARAT improved ~13.9% (video-games) and ~9.6% (traditional), large effect sizes; MAL–Quantity improved from pre to follow-up (medium–large effects). BBT change was not significant. Among participants training standing with video-games, FRT increased but did not reach significance. Follow-up adherence was higher in the video-games arm; no adverse events reported.

3D: three-dimensional; ABILHAND: manual ability questionnaire; ADL: activities of daily living; ARAT: Action Research Arm Test; BBS: Berg Balance Scale; BBT: Box and Block Test; CAHAI: Chedoke Arm and Hand Activity Inventory; CI: confidence interval; EMG: electromyography; EQ-5D: EuroQol 5-Dimensions; FIM: Functional Independence Measure; FMA-UE: Fugl-Meyer Assessment Upper Extremity; FRT: Functional Reach Test; GRASP: Graded Repetitive Arm Supplementary Program; HoVRS: Home-based Virtual Rehabilitation System; HMD: head-mounted display; IMU: inertial measurement unit; MAL: Motor Activity Log; PC: personal computer; QUEST: Quebec User Evaluation of Satisfaction with Assistive Technology; RCT: randomized controlled trial; RGS: Rehabilitation Gaming System; RGB-D: red green blue plus depth; SE: standard error; SIS: Stroke Impact Scale; SUS: System Usability Scale; TUG: Timed Up and Go; VERGE: Virtual Environment for Rehabilitative Gaming Exercises; VAS: Visual Analogue Scale; WMFT: Wolf Motor Function Test; VR: virtual reality; *platform commercially available.

Table S3. Barriers for clinic-based VR exergaming.

Study	Recruitment / engagement barriers	Technical barriers / complexity	Patient-related barriers	Therapist / resource barriers
Korkusuz et al. (2025)[25]	NI	Kinect required with ~2.5 m clear space to the sensor; a sensor perception test for each patient; stance rules (feet planted, avoid arm swing); per-patient difficulty adjustments.	Bias toward milder ambulators: allowed knee hyperextension; MMSE ≥24; first-ever stroke; able to walk ≥50 m; ages 51–70; spasticity MAS 1–2.	Additional therapist time needed for calibration, monitoring posture and safety, and adjusting difficulty.
Lesmana et al., 2024[2]	NI	NI	Initial exhaustion at the beginning of exercise sessions was reported; otherwise safe (no adverse events; RPE ≤14).	NI
Peláez-Vélez et al. (2023)[3]	Two dropouts before randomization: one felt VR was "a waste of time"; one had a two-week nonattendance.	Multi-device networked setup: Oculus Quest 2 HMD + Kinect 360 v1 sensor + PC with Blexer + router using Virtual Desktop streaming; tailored calibration for each patient; of 4 available mini-games, only 3 were used (the fourth was too difficult).	Inclusion required ability to stand, active mobility in at least one upper extremity, and ability to follow simple instructions; a chair was placed next to the patient for possible headache, dizziness, or rest; no specific adverse events were reported.	Continuous therapist supervision: a physical therapist stood behind the patient monitoring the PC; targets and time limits were set by the therapist; VR was always delivered after conventional neurological physical therapy (resource-intensive schedule).
Sultan et al. (2023)[27]	NI	NI	NI	Kinect games were delivered under supervision in a participant-friendly, safe environment.
Ali et al. (2022)[28]	NI	NI	Excluded participants with visual, auditory, speech, or cognitive impairments; excluded those with musculoskeletal gait disorders or cardiovascular complications; required MoCA > 26 and minimal spasticity (bias toward milder cases).	NI
Ul et al. (2022)[30]	NI	Kinect required with proper spacing and camera placement; per-session	Excluded participants with significant cognitive or visual	NI

		sensor positioned at 1.5–2 m; dedicated room setup; no device failures reported.	impairments and severe spasticity; required ability to read and provide consent (bias toward milder cases).	
Malik & Masood (2021) [34]	NI	NI	Excluded participants with severe spasticity or cognitive issues; required ability to stand unaided (selection constraints).	NI
Marques-Sule et al. (2021)[33]	NI	NI	Ensuring appropriate functionality and capacity to learn exercises are prerequisites for safe home Wii use (may limit who can use it).	Home use would require caregiver training and potentially home adaptations or technical aids; safety at home depends on these factors; no device failures were reported.
Norouzi-Gheidari et al. (2020)[10]	NI	NI	NI	Therapist presence was required every session; therapists adjusted settings and monitored progress.
Ahmad et al. (2019)[11]	Intrinsic motivation after the intervention favored the control group on the IMI (authors note lower motivation in the VR group, suggesting an engagement challenge); attendance was limited to once-weekly clinic visits.	NI	Required chronic stroke status and ability to grasp and operate the handlebar (bias toward milder upper-limb impairment and adequate comprehension).	NI
Henrique et al. (2019)[12]	NI	NI	Inclusion required MMSE ≥ 19; excluded participants with global aphasia or severe spasticity (MAS 3–4).	NI
Hung et al. (2019)[13]	Engagement was monitored with PPS (median 5.25 vs 5.00, not significant).	Kinect v1 setup required; game parameters tuned per patient; system detected compensations; accelerometers used at sessions 12 and 24.	Transient upper-extremity soreness was reported in K2S; no serious adverse events occurred; inclusion required MMSE > 20 and active proximal upper extremity (bias toward milder deficits).	Therapist supervision was provided every session.
Kannan et al. (2019)[35]	NI	NI	Required independent standing for ≥5 min, MMSE ≥25, and no aphasia;	NI

			excluded participants with osteoporosis (heel T-score < –2.0), seizures, or major comorbid neurological conditions (bias toward milder, safer cases).	
Miranda et al. (2019)[36]	NI	NI	Required ability to independently walk 10 m, stand for 60 s, and step up one stair; excluded those with severe cognitive impairment (MoCA < 20); participants had no prior platform-game experience (implying initial unfamiliarity).	Only the first session included physiotherapist verbal and manual guidance; sessions 2 and 3 were unguided, reducing ongoing supervision for technique correction.
Subramaniam & Bhatt (2019)[37]	Of 18 interested, 3 were excluded at phone screening; 1 dropped out mid-study due to commute difficulty; 1 was excluded from analysis for technical data-collection problems. Small final n = 13.	NI	Required independent standing ≥5 min; cognitive screening (SOMC ≥ 10); excluded participants with recent surgery, shoulder pain, or significant neurological/musculoskeletal/cardiac issues (skewed to milder, safer cases).	Contact-guard assistance and a gait belt were used each session; high visit burden (20 lab sessions over 6 weeks, with warm-up/cool-down and song progression).
Carregosa et al. (2018)[38]	Attrition: of 15 participants who started the Wii program, only 5 completed the 2-month follow-up.	NI	Required ability to hold the controller, walk unaided, and understand game tasks (selection toward milder deficits).	Sessions were supervised at the school clinic; therapists planned game selection and adjusted difficulty.
Lupo et al. (2018)[14]	Small sample (n=15; 9 intervention / 6 control); the RIABLO group showed higher participation than control (suggesting an engagement benefit rather than a barrier).	Multi-sensor setup: 3 IMUs + force platform, Bluetooth 50 Hz, straps and calibration; required dedicated PC software and space for static/dynamic stance tasks; stabilometric platform used for outcomes.	Inclusion biased toward milder subacute cases: able to walk with supervision, MMSE ≥ 23; excluded those with severe neglect or limiting orthopedic issues.	NI
Perez-Marcos et al. (2017)[16]	NI	One participant was sensitive to screen exposure (headache led to shorter sessions); colored markers	Occasional early fatigue (session 2) with rapid dose increase; two cases of shoulder pain (unrelated to device); overall low pain and stress	A therapist was needed each session to select and progress tasks.

		and IMUs required setup; otherwise, high system efficiency.	across sessions; no serious adverse events.	
Standen et al. (2017)[59]	Low recruitment and consent rates; high dropout due to health issues, absences, or competing commitments.	Frequent technical problems; high setup and training requirements.	NI	High ongoing support and training needed from therapists/researchers.
Stockley et al. (2017)[17]	Slow recruitment (small eligible pool, ~2/month); all completed intervention (no dropouts)	Software freezing; lack of structure/progression in games; occasional sensor issues; required standby supervision	Fatigue; energy/concentration demands; frustration if unable to complete games. Games not tailored to all deficits; desire for more specific/progressive content	Increased staff supervision/support needed due to technical challenges and system setup
Lloréns et al. (2015)[18]	NI	NI	Two discontinuations occurred during the trial due to recurrent stroke (one per group); no session-related side effects were reported.	NI
da Silva Ribeiro et al. (2015)[40]	NI	Dedicated setup: requires Nintendo Wii + multimedia projector in a 20 m² room (image projected at ~1.2 m height); session-by-session game selection and difficulty tuning; 1-min rests between games; no device failures were noted.	Eligibility skewed to milder cases: participants must be able to ambulate and hold the controller; excluded were those with hemineglect, Pusher syndrome, intellectual disability limiting game understanding, or orthopedic disorders limiting task performance; ≥6 months post-stroke.	NI
Hung et al. (2014)[41]	NI	Wii Balance Board with a safety walker in front; game selection each session; initial compensatory movements required coaching; some spasticity was provoked by the Basic Step game; Tetrax posturography used for testing.	Inclusion required ability to walk 10 m, understand instructions, and view a TV; excluded participants with neglect, major visual field loss, receptive aphasia, or other neurological disorders (skewed to milder cases).	Direct occupational therapy supervision twice weekly; therapists adjusted game choices and difficulty and provided safety guarding.
Crosbie et al. (2012)[20]	NI	Used a head-mounted display (HMD) with electromagnetic tracking; pacemakers were excluded (potential interference); transient	Excluded participants with severe cognitive or visuospatial deficits.	NI

		dizziness and headache occurred in two VR participants.		
Reinthal et al. (2012)[44]	NI	NI	About 50% needed hands-on assistance during sessions; inclusion required unsupported sitting balance and the ability to learn the game within 3 sessions.	A clinician spent ~10 minutes per session directing game selection and progression; sessions were delivered by trained support staff/students under clinician oversight.
Singh et al. (2013)[43]	50 were screened; 28 were analyzed; dropouts included refusals, no-shows, transportation problems, and one fall at home (outside of sessions).	Wii Balance Board and Xbox Kinect required; one-to-one supervision during gaming; progression rules (e.g., medal threshold for game change); separate ProBalance board used for static balance testing; no device failures were reported.	Inclusion skewed to milder/safer cases: age ≥55, independent walking, ability to stand ≥30 min, MMSE ≥17; excluded medications affecting balance and serious comorbid illnesses (limits generalizability).	NI
Kim et al. (2009)[22]	NI	NI	Inclusion skewed to milder chronic cases: ≥1 year post-stroke, able to stand 30 min and walk 30 m; excluded those with severe visual neglect or cognitive impairment.	Therapist time was required for both CPT and VR sessions; CPT was individualized; VR progression (adding weights/speed) required supervision; authors noted difficulty in standardizing treatment duration in the hospital setting.
Broeren et al. (2008)[23]	Initial hesitancy toward 3D computer games; all participants were novice game users; willingness improved after exposure.	Occasional game startup failures, resolved via remote instructions; tele-support (Skype) used for troubleshooting.	Some games were perceived as difficult (Archery, Simon) while others were easy (Bingo, Fish Tank); all participants learned the system quickly.	Center staff had no prior VR experience.

NI: no information; 3D: three-dimensional; CPT: conventional physical therapy; FRT: Functional Reach Test; HMD: head-mounted display; IMI: Intrinsic Motivation Inventory; IMU: inertial measurement unit; MMSE: Mini-Mental State Examination; MoCA: Montreal Cognitive Assessment; PC: personal computer; PPS: Pittsburgh Participation Scale; PT: physical therapy; RPE: Rating of Perceived Exertion; SOMC: Short Orientation-Memory-Concentration Test; VR: virtual reality.

Table S4. Barriers for home-based VR exergaming.

Study	Recruitment / engagement barriers	Technical barriers / complexity	Patient-related barriers	Therapist / resource barriers
Ase et al. (2025)[45]	NI	NI	Glove requires a minimum active range of motion; it is more practical for mild–moderate paresis (authors note VR without external force suits those with some movement).	Weekly outpatient OT needed for calibration and task adjustment alongside home program (resource demand).
Sheehy et al. (2025)[46]	NI	Tight space in 5 homes (<2.0 m) affected tracking; Kinect tracking glitches; OS updates broke software temporarily; one TV required HDMI-to-AV (poor image); reliance on Wi-Fi	One low-motivation participant; some games frustrating/boring; fatigue if done same day as other therapies; 2 had difficulty understanding SIS/CIQ; one had difficulty using the computer	Initial home setup/training by PT; weekly phone support; remote troubleshooting; study partner required for safety/technical help
Fluet et al. (2024)[47]	Dropouts 5/33; one reported not enjoying the games	Setup/play difficulties cited by 4 dropouts; among completers, tech support calls 0–11 (most ≤5); issues: ISP/internet, OS updates, camera; 4 subjects required in-person tech visit	Inclusion required MoCA ≥22 and adequate visual field; some needed mechanical supports to keep hand in Leap Motion workspace (noted as setup need, not a safety AE)	1–6 in-person follow-ups per subject across 12 weeks to swap/adjust games and parameters
Lu et al. (2024)[48]	NI	Requires Kinect V2 sensor, mini-PC, router, and screen; high LCD brightness caused glare and overheating (2.3°C/h at 31°C ambient)	NI	NI
Desai et al. (2023)[49]	Retention: 70% at 1 mo; 50% at 3 mo; satisfaction high; some participants withdrew	Home install (laptop + Kinect); occasional telephone tech assistance	NI	One-time clinic onboarding; remote check-ins; no ongoing supervised sessions
Chen et al. (2022)[50]	NI	NI	NI	Initial onboarding by therapist; assessments every 14 days
Ellis et al. (2022)[51]	4 of 12 did not complete: 2 withdrawn due to inability to contact or declining measurement visits; baseline schedules often slipped	One participant withdrew after baseline because of device challenges (later resolved by developer); platform reliability ≈95% of	NI	Weekly in-person researcher visits for troubleshooting and progress measures

	because of holidays and commitments	intervention days; Kinect v2 was deprecated during the project, requiring migration to Azure Kinect; home setup requires specific space and sensor placement		
Kilbride et al. (2022)[52]	30 recruited over 5 months; 3 withdrew; 90% retention at 8 and 12 weeks; only 26.7% achieved target ≥225 min/week; motivation flagged for more game variety in interviews	9/30 (30%) needed technical support (17 calls, 15 home visits; median 13 min/call, 35 min/visit); most required one training visit only (mean 95 min)	Some with more severe upper-limb impairment needed carer help for setup; muscle fatigue/upper-limb pain/eye strain were common mild AEs; fatigue (FSS-7) unchanged	NI
Jonsdottir et al. (2021)[54]	NI	NI	Inclusion required MMSE ≥20 and ability to stand 30 s	Weekly remote adjustments to program
Lansberg et al(2021)[53]	NI	Glove fit dissatisfaction (3/20); games sometimes too easy (2/20)	Inclusion required minimum active wrist/finger movement (MRC thresholds); older age did not reduce usage	Initial onboarding; phone support (weeks 2 & 6); one clinic visit at week 4 for troubleshooting/progression
Thielbar et al. (2020)[55]	NI	Home install of laptop+Kinect; remote server/VPN; scheduling for multi-user sessions; occasional remote tech assistance	Excluded significant vision/cognitive deficits; moderate UE impairment only	NI
Qiu et al. (2020)[56]	NI	6 total technical issues across cohort (most fixed remotely); requires home internet; some homes needed mounts/stands; Leap Motion workspace positioning occasionally challenging	Many required arm support early; severe impairment (UEFMA <15) not suitable; minimal computer skills acceptable	Initial home setup by PT and engineer
Borstad et al. (2018)[57]	Engagement with transfer package was low; in-game MAL completed ~53% of planned prompts; mitt adherence poor; 1 of 17 did not complete study	Kinect plus glove sensors required setup and therapist customization; one case of shoulder pain until game parameters were adjusted	Mitt disliked; fatigue possible at high target dose; cognitive or memory issues may limit carryover	NI
Golla et al. (2018)[64]	NI	Setup required home visit (to create a structured environment) and weekly progress monitoring; remote	NI	24/7 remote supervision via VNC; diaries for self-monitoring; required carer support and accident insurance

		management initially challenging; diaries needed; one participant sustained a foot injury		(adding staff time and logistical burden)
Rand et al. (2016)[66]	Low recruitment: only 16.9% of screened participants enrolled; some participants withdrew when assigned to the VR gaming arm	NI	Participation was limited to those with mild–moderate UE impairment; findings may not generalize to more disabled patients	Required family/caregiver involvement at home and OT follow-up; staffing/time and travel logistics were significant burdens
Wittmann et al. (2016)[61]	NI	NI	Severe impairment → lower usage; risk of trunk compensation (generally small)	NI
Zondervan et al. (2016)[62]	NI	Home kit required laptop + MusicGlove; common user issues: launching app (double-click), trackpad cursor control, exiting app, powering laptop; two cases of missing usage logs due to technical errors	Inclusion/exclusion biased toward milder chronic cases: age <75, BBT ≥1, pain ≤5/10, MAS ≤3, adequate sensation, no severe visual deficits/neglect/apraxia; required ability to follow instructions	NI

NI: no information; AE: adverse event; AEs: adverse events; ARAT: Action Research Arm Test; AV: audio video; BBT: Box and Block Test; CIQ: Community Integration Questionnaire; FSS-7: Fatigue Severity Scale, 7-item version; HDMI: High-Definition Multimedia Interface; ISP: internet service provider; LCD: liquid crystal display; MAL: Motor Activity Log; MMSE: Mini-Mental State Examination; MoCA: Montreal Cognitive Assessment; MRC: Medical Research Council; OS: operating system; OT: occupational therapy; PC: personal computer; PT: physical therapy; SIS: Stroke Impact Scale; TV: television; UE: upper extremity; UEFMA: Upper Extremity Fugl-Meyer Assessment; VNC: Virtual Network Computing; VPN: virtual private network; VR: virtual reality; Wi-Fi: wireless networking.

References

1. Jo, S.; Jang, H.; Kim, H.; Song, C. 360° Immersive Virtual Reality-Based Mirror Therapy for Upper Extremity Function and Satisfaction among Stroke Patients: A Randomized Controlled Trial. *Eur. J. Phys. Rehabil. Med.* 2024, *60*, 207–215, doi:10.23736/S1973-9087.24.08275-3.
2. Lesmana, I.P.D.; Destarianto, P.; Widiawan, B.; Suryana, A.L.; Hossain, F.S. Effectiveness of Virtual Reality Cycling Exercise towards the Motoric and Cardiorespiratory Functions of Post-Stroke Patients. *Physiother. Q.* 2024, *32*, 107–114, doi:10.5114/pq.2024.126951.
3. Peláez-Vélez, F.-J.; Eckert, M.; Gacto-Sánchez, M.; Martínez-Carrasco, Á. Use of Virtual Reality and Videogames in the Physiotherapy Treatment of Stroke Patients: A Pilot Randomized Controlled Trial. *Int. J. Environ. Res. Public. Health* 2023, *20*, 4747, doi:10.3390/ijerph20064747.
4. Hsu, H.-Y.; Kuo, L.-C.; Lin, Y.-C.; Su, F.-C.; Yang, T.-H.; Lin, C.-W. Effects of a Virtual Reality-Based Mirror Therapy Program on Improving Sensorimotor Function of Hands in Chronic Stroke Patients: A Randomized Controlled Trial. *Neurorehabil. Neural Repair* 2022, *36*, 335–345, doi:10.1177/15459683221081430.
5. Junata, M.; Cheng, K.C.-C.; Man, H.S.; Lai, C.W.-K.; Soo, Y.O.-Y.; Tong, R.K.-Y. Kinect-Based Rapid Movement Training to Improve Balance Recovery for Stroke Fall Prevention: A Randomized Controlled Trial. *J. NeuroEngineering Rehabil.* 2021, *18*, 150, doi:10.1186/s12984-021-00922-3.
6. Escalante-Gonzalbo, A.M.; Ramírez-Graullera, Y.S.; Pasantes, H.; Aguilar-Chalé, J.J.; Sánchez-Castillo, G.I.; Escutia-Macedo, X.A.; Briseño-Soriano, T.M.; Franco-Castro, P.; Estrada-Rosales, A.L.; Vázquez-Abundes, S.E.; et al. Safety, Feasibility, and Acceptability of a New Virtual Rehabilitation Platform: A Supervised Pilot Study. *Rehabil. Process Outcome* 2021, *10*, 11795727211033279, doi:10.1177/11795727211033279.
7. Xu, Y.; Tong, M.; Ming, W.-K.; Lin, Y.; Mai, W.; Huang, W.; Chen, Z. A Depth Camera–Based, Task-Specific Virtual Reality Rehabilitation Game for Patients With Stroke: Pilot Usability Study. *JMIR Serious Games* 2021, *9*, e20916, doi:10.2196/20916.
8. Johnson, L.; Bird, M.-L.; Muthalib, M.; Teo, W.-P. An Innovative STRoke Interactive Virtual thErapy (STRIVE) Online Platform for Community-Dwelling Stroke Survivors: A Randomized Controlled Trial. *Arch. Phys. Med. Rehabil.* 2020, *101*, 1131–1137, doi:10.1016/j.apmr.2020.03.011.
9. Lee, H.-S.; Lim, J.-H.; Jeon, B.-H.; Song, C.-S. Non-Immersive Virtual Reality Rehabilitation Applied to a Task-Oriented Approach for Stroke Patients: A Randomized Controlled Trial. *Restor. Neurol. Neurosci.* 2020, *38*, 165–172, doi:10.3233/RNN-190975.
10. Norouzi-Gheidari, N.; Hernandez, A.; Archambault, P.S.; Higgins, J.; Poissant, L.; Kairy, D. Feasibility, Safety and Efficacy of a Virtual Reality Exergame System to Supplement Upper Extremity Rehabilitation Post-Stroke: A Pilot Randomized Clinical Trial and Proof of Principle. *Int. J. Environ. Res. Public. Health* 2020, *17*, 113, doi:10.3390/ijerph17010113.
11. Ahmad, M.A.; Singh, D.K.A.; Mohd Nordin, N.A.; Hooi Nee, K.; Ibrahim, N. Virtual Reality Games as an Adjunct in Improving Upper Limb Function and General Health among Stroke Survivors. *Int. J. Environ. Res. Public. Health* 2019, *16*, 5144, doi:10.3390/ijerph16245144.
12. Henrique, P.P.B.; Colussi, E.L.; De Marchi, A.C.B. Effects of Exergame on Patients' Balance and Upper Limb Motor Function after Stroke: A Randomized Controlled Trial. *J. Stroke Cerebrovasc. Dis. Off. J. Natl. Stroke Assoc.* 2019, *28*, 2351–2357, doi:10.1016/j.jstrokecerebrovasdis.2019.05.031.
13. Hung, J.-W.; Chou, C.-X.; Chang, Y.-J.; Wu, C.-Y.; Chang, K.-C.; Wu, W.-C.; Howell, S. Comparison of Kinect2Scratch Game-Based Training and Therapist-Based Training for the Improvement of Upper Extremity Functions of Patients with Chronic Stroke: A Randomized Controlled Single-Blinded Trial. *Eur. J. Phys. Rehabil. Med.* 2019, *55*, 542–550, doi:10.23736/S1973-9087.19.05598-9.
14. Lupo, A.; Cinnera, A.M.; Pucello, A.; Iosa, M.; Coiro, P.; Personeni, S.; Gimigliano, F.; Iolascon, G.; Paolucci, S.; Morone, G. Effects on Balance Skills and Patient Compliance of Biofeedback Training with Inertial Measurement Units and Exergaming in Subacute Stroke: A Pilot Randomized Controlled Trial. *Funct. Neurol.* 2018, *33*, 131–136.
15. Noveletto, F.; Soares, A.V.; Mello, B.A.; Sevegnani, C.N.; Eichinger, F.L.F.; Hounsell, M.D.S.; Bertemes-Filho, P. Biomedical Serious Game System for Balance Rehabilitation of Hemiparetic Stroke Patients. *IEEE Trans. Neural Syst. Rehabil. Eng.* 2018, *26*, 2179–2188, doi:10.1109/TNSRE.2018.2876670.
16. Perez-Marcos, D.; Chevalley, O.; Schmidlin, T.; Garipelli, G.; Serino, A.; Vuadens, P.; Tadi, T.; Blanke, O.; Millán, J.D.R. Increasing Upper Limb Training Intensity in Chronic Stroke Using Embodied Virtual Reality: A Pilot Study. *J. Neuroengineering Rehabil.* 2017, *14*, 119, doi:10.1186/s12984-017-0328-9.
17. Stockley, R.C.; O'Connor, D.A.; Smith, P.; Moss, S.; Allsop, L.; Edge, W. A Mixed Methods Small Pilot Study to Describe the Effects of Upper Limb Training Using a Virtual Reality Gaming System in People with Chronic Stroke. *Rehabil. Res. Pract.* 2017, *2017*, 9569178, doi:10.1155/2017/9569178.

18. Lloréns, R.; Gil-Gómez, J.-A.; Alcañiz, M.; Colomer, C.; Noé, E. Improvement in Balance Using a Virtual Reality-Based Stepping Exercise: A Randomized Controlled Trial Involving Individuals with Chronic Stroke. *Clin. Rehabil.* 2015, *29*, 261–268, doi:10.1177/0269215514543333.
19. Tsoupikova, D.; Stoykov, N.S.; Corrigan, M.; Thielbar, K.; Vick, R.; Li, Y.; Triandafilou, K.; Preuss, F.; Kamper, D. Virtual Immersion for Post-Stroke Hand Rehabilitation Therapy. *Ann. Biomed. Eng.* 2015, *43*, 467–477, doi:10.1007/s10439-014-1218-y.
20. Crosbie, J.; Lennon, S.; McGoldrick, M.; McNeill, M.; McDonough, S. Virtual Reality in the Rehabilitation of the Arm after Hemiplegic Stroke: A Randomized Controlled Pilot Study. *Clin. Rehabil.* 2012, *26*, 798–806, doi:10.1177/0269215511434575.
21. Levin, M.F.; Snir, O.; Liebermann, D.G.; Weingarden, H.; Weiss, P.L. Virtual Reality Versus Conventional Treatment of Reaching Ability in Chronic Stroke: Clinical Feasibility Study. *Neurol. Ther.* 2012, *1*, 3, doi:10.1007/s40120-012-0003-9.
22. Kim, J.H.; Jang, S.H.; Kim, C.S.; Jung, J.H.; You, J.H. Use of Virtual Reality to Enhance Balance and Ambulation in Chronic Stroke: A Double-Blind, Randomized Controlled Study. *Am. J. Phys. Med. Rehabil.* 2009, *88*, 693, doi:10.1097/PHM.0b013e3181b33350.
23. Broeren, J.; Claesson, L.; Goude, D.; Rydmark, M.; Sunnerhagen, K.S. Virtual Rehabilitation in an Activity Centre for Community-Dwelling Persons with Stroke: The Possibilities of 3-Dimensional Computer Games. *Cerebrovasc. Dis.* 2008, *26*, 289–296, doi:10.1159/000149576.
24. Yang, Y.-R.; Tsai, M.-P.; Chuang, T.-Y.; Sung, W.-H.; Wang, R.-Y. Virtual Reality-Based Training Improves Community Ambulation in Individuals with Stroke: A Randomized Controlled Trial. *Gait Posture* 2008, *28*, 201–206, doi:10.1016/j.gaitpost.2007.11.007.
25. Korkusuz, S.; Taşkın, G.; Korkusuz, B.S.; Özen, M.S.; Yürük, Z.Ö. Examining the Effects of Non-Immersive Virtual Reality Game-Based Training on Knee Hyperextension Control and Balance in Chronic Stroke Patients: A Single-Blind Randomized Controlled Study. *Neurol. Sci. Off. J. Ital. Neurol. Soc. Ital. Soc. Clin. Neurophysiol.* 2025, *46*, 1267–1275, doi:10.1007/s10072-024-07830-z.
26. Farahmand, S.; Ghasemi, M.; Basiri, K.; Ghasemi, E. Investigating the Effects of Nintendo Wii on Ankle Spasticity in Patients with Stroke: A Randomized Clinical Trial. *J. Mod. Rehabil.* 2024, *18*, 480–488, doi:10.18502/jmr.v18i4.16917.
27. Sultan, N.; Khushnood, K.; Qureshi, S.; Altaf, S.; Khan, M.K.; Malik, A.N.; Mehmood, R.; Awan, M.M.A. Effects of Virtual Reality Training Using Xbox Kinect on Balance, Postural Control, and Functional Independence in Subjects with Stroke. *Games Health J.* 2023, *12*, 440–444, doi:10.1089/g4h.2022.0193.
28. Ali, A.; Shendy, W.; Hassan, A.; Abdelmonem, K.; El Khatib, A. The Impact of Virtual Reality Training with a Cognitive Load on Falling in Stroke Cases. *NeuroQuantology* 2022, *20*, 90–97, doi:10.14704/NQ.2022.20.15.NQ88007.
29. Anwar, N.; Karimi, H.; Ahmad, A.; Gilani, S.A.; Khalid, K.; Aslam, A.S.; Hanif, A. Virtual Reality Training Using Nintendo Wii Games for Patients With Stroke: Randomized Controlled Trial. *JMIR Serious Games* 2022, *10*, e29830, doi:10.2196/29830.
30. Ul, A.Q.; Khan, S.; Ishtiaq, S.; Alsaied, A.; Liu, T.; Wang, J. Therapeutic Benefits of Xbox Kinect Training on Upper Limb Motor Function in Chronic Stroke Patients. In Proceedings of the 2022 8th International Conference on Virtual Reality (ICVR); May 2022; pp. 142–146.
31. Anwar, N.; Karimi, H.; Ahmad, A.; Mumtaz, N.; Saqulain, G.; Gilani, S.A. A Novel Virtual Reality Training Strategy for Poststroke Patients: A Randomized Clinical Trial. *J. Healthc. Eng.* 2021, *2021*, 6598726, doi:10.1155/2021/6598726.
32. Ain, Q.U.; Khan, S.; Ilyas, S.; Yaseen, A.; Tariq, I.; Liu, T.; Wang, J. Additional Effects of Xbox Kinect Training on Upper Limb Function in Chronic Stroke Patients: A Randomized Control Trial. *Healthc. Basel Switz.* 2021, *9*, 242, doi:10.3390/healthcare9030242.
33. Marques-Sule, E.; Arnal-Gómez, A.; Buitrago-Jiménez, G.; Suso-Martí, L.; Cuenca-Martínez, F.; Espí-López, G.V. Effectiveness of Nintendo Wii and Physical Therapy in Functionality, Balance, and Daily Activities in Chronic Stroke Patients. *J. Am. Med. Dir. Assoc.* 2021, *22*, 1073–1080, doi:10.1016/j.jamda.2021.01.076.
34. Malik, A.N.; Masood, T. Task-Oriented Training and Exer-Gaming for Improving Mobility after Stroke: A Randomized Trial. *JPMA J. Pak. Med. Assoc.* 2021, *71*, 186–190, doi:10.47391/JPMA.560.
35. Kannan, L.; Vora, J.; Bhatt, T.; Hughes, S.L. Cognitive-Motor Exergaming for Reducing Fall Risk in People with Chronic Stroke: A Randomized Controlled Trial. *NeuroRehabilitation* 2019, *44*, 493–510, doi:10.3233/NRE-182683.
36. Miranda, C.S.; Oliveira, T. de P.; Gouvêa, J.X.M.; Perez, D.B.; Marques, A.P.; Piemonte, M.E.P. Balance Training in Virtual Reality Promotes Performance Improvement but Not Transfer to Postural Control in People with Chronic Stroke. *Games Health J.* 2019, *8*, 294–300, doi:10.1089/g4h.2018.0075.

37. Subramaniam, S.; Bhatt, T. Dance-Based Exergaming for Upper Extremity Rehabilitation and Reducing Fall-Risk in Community-Dwelling Individuals with Chronic Stroke. A Preliminary Study. *Top. Stroke Rehabil.* 2019, *26*, 565–575, doi:10.1080/10749357.2019.1625545.
38. Carregosa, A.A.; Santos, L.R.A. dos; Masruha, M.R.; Coêlho, M.L. da S.; Machado, T.C.; Souza, D.C.B.; Passos, G.L.L.; Fonseca, E.P.; Ribeiro, N.M. da S.; Melo, A. de S. Virtual Rehabilitation through Nintendo Wii in Poststroke Patients: Follow-Up. *J. Stroke Cerebrovasc. Dis.* 2018, *27*, 494–498, doi:10.1016/j.jstrokecerebrovasdis.2017.09.029.
39. Fonseca, E.P. da; Silva, N.M.R. da; Pinto, E.B. Therapeutic Effect of Virtual Reality on Post-Stroke Patients: Randomized Clinical Trial. *J. Stroke Cerebrovasc. Dis.* 2017, *26*, 94–100, doi:10.1016/j.jstrokecerebrovasdis.2016.08.035.
40. da Silva Ribeiro, N.M.; Ferraz, D.D.; Pedreira, É.; Pinheiro, Í.; da Silva Pinto, A.C.; Neto, M.G.; Dos Santos, L.R.A.; Pozzato, M.G.G.; Pinho, R.S.; Masruha, M.R. Virtual Rehabilitation via Nintendo Wii® and Conventional Physical Therapy Effectively Treat Post-Stroke Hemiparetic Patients. *Top. Stroke Rehabil.* 2015, *22*, 299–305, doi:10.1179/1074935714Z.0000000017.
41. Hung, J.-W.; Chou, C.-X.; Hsieh, Y.-W.; Wu, W.-C.; Yu, M.-Y.; Chen, P.-C.; Chang, H.-F.; Ding, S.-E. Randomized Comparison Trial of Balance Training by Using Exergaming and Conventional Weight-Shift Therapy in Patients with Chronic Stroke. *Arch. Phys. Med. Rehabil.* 2014, *95*, 1629–1637, doi:10.1016/j.apmr.2014.04.029.
42. Fritz, S.L.; Peters, D.M.; Merlo, A.M.; Donley, J. Active Video-Gaming Effects on Balance and Mobility in Individuals with Chronic Stroke: A Randomized Controlled Trial. *Top. Stroke Rehabil.* 2013, *20*, 218–225, doi:10.1310/tsr2003-218.
43. Singh, D.K.A.; Mohd Nordin, N.A.; Aziz, N.A.A.; Lim, B.K.; Soh, L.C. Effects of Substituting a Portion of Standard Physiotherapy Time with Virtual Reality Games among Community-Dwelling Stroke Survivors. *BMC Neurol.* 2013, *13*, 199, doi:10.1186/1471-2377-13-199.
44. Reinthal, A.; Szirony, K.; Clark, C.; Swiers, J.; Kellicker, M.; Linder, S. ENGAGE: Guided Activity-Based Gaming in Neurorehabilitation after Stroke: A Pilot Study. *Stroke Res. Treat.* 2012, *2012*, 784232, doi:10.1155/2012/784232.
45. Ase, H.; Honaga, K.; Tani, M.; Takakura, T.; Wada, F.; Murakami, Y.; Isayama, R.; Tanuma, A.; Fujiwara, T. Effects of Home-Based Virtual Reality Upper Extremity Rehabilitation in Persons with Chronic Stroke: A Randomized Controlled Trial. *J. Neuroengineering Rehabil.* 2025, *22*, 20, doi:10.1186/s12984-025-01564-5.
46. Sheehy, L.; Taillon-Hobson, A.; Sveistrup, H.; Bilodeau, M.; Yang, C.; Welch, V.; Finestone, H. Home-Based Nonimmersive Virtual Reality Training After Discharge From Inpatient or Outpatient Stroke Rehabilitation: Parallel Feasibility Randomized Controlled Trial. *JMIR Rehabil. Assist. Technol.* 2025, *12*, e64729, doi:10.2196/64729.
47. Fluet, G.; Qiu, Q.; Gross, A.; Gorin, H.; Patel, J.; Merians, A.; Adamovich, S. The Influence of Scaffolding on Intrinsic Motivation and Autonomous Adherence to a Game-Based, Sparsely Supervised Home Rehabilitation Program for People with Upper Extremity Hemiparesis Due to Stroke. A Randomized Controlled Trial. *J. Neuroengineering Rehabil.* 2024, *21*, 143, doi:10.1186/s12984-024-01441-7.
48. Lu, H.-Y.; Wang, X.; Hu, C.; Lau, C.C.-Y.; Tong, R.K.-Y. Home-Based Guidance Training System with Interactive Visual Feedback Using Kinect on Stroke Survivors with Moderate to Severe Motor Impairment. *J. NeuroEngineering Rehabil.* 2024, *21*, 189, doi:10.1186/s12984-024-01479-7.
49. Desai, K.; Prabhakaran, B.; Ifejika, N.; Annaswamy, T.M. Personalized 3D Exergames for In-Home Rehabilitation after Stroke: A Pilot Study. *Disabil. Rehabil. Assist. Technol.* 2023, *18*, 704–713, doi:10.1080/17483107.2021.1913518.
50. Chen, C.-H.; Kreidler, T.; Ochsenfahrt, A. Rehago - A Home-Based Training App Using Virtual Reality to Improve Functional Performance of Stroke Patients with Mirror Therapy and Gamification Concept: A Pilot Study. *Stud. Health Technol. Inform.* 2022, *292*, 91–95, doi:10.3233/SHTI220330.
51. Ellis, F.; Hancock, N.; Kennedy, N.; Clark, A.; Wells, J.; Chandler, E.; Payne, D.; Pomeroy, V.M. Consideration-of-Concept of EvolvRehab-Body for Upper Limb Virtual Rehabilitation at Home for People Late after Stroke. *Physiotherapy* 2022, *116*, 97–107, doi:10.1016/j.physio.2022.03.004.
52. Kilbride, C.; Scott, D.J.M.; Butcher, T.; Norris, M.; Warland, A.; Anokye, N.; Cassidy, E.; Baker, K.; Athanasiou, D.A.; Singla-Buxarrais, G.; et al. Safety, Feasibility, Acceptability and Preliminary Effects of the Neurofenix Platform for Rehabilitation via HOMe Based Gaming Exercise for the Upper-Limb Post Stroke (RHOMBUS): Results of a Feasibility Intervention Study. *BMJ Open* 2022, *12*, e052555, doi:10.1136/bmjopen-2021-052555.
53. Lansberg, M.G.; Legault, C.; MacLellan, A.; Parikh, A.; Muccini, J.; Mlynash, M.; Kemp, S.; Buckwalter, M.S.; Flavin, K. Home-Based Virtual Reality Therapy for Hand Recovery after Stroke. *PM&R* 2022, *14*, 320–328, doi:10.1002/pmrj.12598.

54. Jonsdottir, J.; Baglio, F.; Gindri, P.; Isernia, S.; Castiglioni, C.; Gramigna, C.; Palumbo, G.; Pagliari, C.; Di Tella, S.; Perini, G.; et al. Virtual Reality for Motor and Cognitive Rehabilitation From Clinic to Home: A Pilot Feasibility and Efficacy Study for Persons With Chronic Stroke. *Front. Neurol.* 2021, *12*, 601131, doi:10.3389/fneur.2021.601131.
55. Thielbar, K.O.; Triandafilou, K.M.; Barry, A.J.; Yuan, N.; Nishimoto, A.; Johnson, J.; Stoykov, M.E.; Tsoupikova, D.; Kamper, D.G. Home-Based Upper Extremity Stroke Therapy Using a Multiuser Virtual Reality Environment: A Randomized Trial. *Arch. Phys. Med. Rehabil.* 2020, *101*, 196–203, doi:10.1016/j.apmr.2019.10.182.
56. Qiu, Q.; Cronce, A.; Patel, J.; Fluet, G.G.; Mont, A.J.; Merians, A.S.; Adamovich, S.V. Development of the Home Based Virtual Rehabilitation System (HoVRS) to Remotely Deliver an Intense and Customized Upper Extremity Training. *J. NeuroEngineering Rehabil.* 2020, *17*, 155, doi:10.1186/s12984-020-00789-w.
57. Borstad, A.L.; Crawfis, R.; Phillips, K.; Lowes, L.P.; Maung, D.; McPherson, R.; Siles, A.; Worthen-Chaudhari, L.; Gauthier, L.V. In-Home Delivery of Constraint-Induced Movement Therapy via Virtual Reality Gaming. *J. Patient-Centered Res. Rev.* 2018, *5*, 6–17, doi:10.17294/2330-0698.1550.
58. Ballester, B.R.; Nirme, J.; Camacho, I.; Duarte, E.; Rodríguez, S.; Cuxart, A.; Duff, A.; Verschure, P.F.M.J. Domiciliary VR-Based Therapy for Functional Recovery and Cortical Reorganization: Randomized Controlled Trial in Participants at the Chronic Stage Post Stroke. *JMIR Serious Games* 2017, *5*, e15, doi:10.2196/games.6773.
59. Standen, P.J.; Threapleton, K.; Richardson, A.; Connell, L.; Brown, D.J.; Battersby, S.; Platts, F.; Burton, A. A Low Cost Virtual Reality System for Home Based Rehabilitation of the Arm Following Stroke: A Randomised Controlled Feasibility Trial. *Clin. Rehabil.* 2017, *31*, 340–350, doi:10.1177/0269215516640320.
60. Nijenhuis, S.M.; Prange-Lasonder, G.B.; Stienen, A.H.; Rietman, J.S.; Buurke, J.H. Effects of Training with a Passive Hand Orthosis and Games at Home in Chronic Stroke: A Pilot Randomised Controlled Trial. *Clin. Rehabil.* 2017, *31*, 207–216, doi:10.1177/0269215516629722.
61. Wittmann, F.; Held, J.P.; Lambercy, O.; Starkey, M.L.; Curt, A.; Höver, R.; Gassert, R.; Luft, A.R.; Gonzenbach, R.R. Self-Directed Arm Therapy at Home after Stroke with a Sensor-Based Virtual Reality Training System. *J. NeuroEngineering Rehabil.* 2016, *13*, 75, doi:10.1186/s12984-016-0182-1.
62. Zondervan, D.K.; Friedman, N.; Chang, E.; Zhao, X.; Augsburger, R.; Reinkensmeyer, D.J.; Cramer, S.C. Home-Based Hand Rehabilitation after Chronic Stroke: Randomized, Controlled Single-Blind Trial Comparing the MusicGlove with a Conventional Exercise Program. *J. Rehabil. Res. Dev.* 2016, *53*, 457–472, doi:10.1682/JRRD.2015.04.0057.
63. Slijper, A.; Svensson, K.E.; Backlund, P.; Engström, H.; Sunnerhagen, K.S. Computer Game-Based Upper Extremity Training in the Home Environment in Stroke Persons: A Single Subject Design. *J. Neuroengineering Rehabil.* 2014, *11*, 35, doi:10.1186/1743-0003-11-35.
64. Golla, A.; Müller, T.; Wohlfarth, K.; Jahn, P.; Mattukat, K.; Mau, W. Home-Based Balance Training Using Wii Fit™: A Pilot Randomised Controlled Trial with Mobile Older Stroke Survivors. *Pilot Feasibility Stud.* 2018, *4*, 143, doi:10.1186/s40814-018-0334-0.
65. Adie, K.; Schofield, C.; Berrow, M.; Wingham, J.; Humfryes, J.; Pritchard, C.; James, M.; Allison, R. Does the Use of Nintendo Wii SportsTM Improve Arm Function? Trial of WiiTM in Stroke: A Randomized Controlled Trial and Economics Analysis. *Clin. Rehabil.* 2017, *31*, 173–185, doi:10.1177/0269215516637893.
66. Rand, D.; Weingarden, H.; Weiss, R.; Yacoby, A.; Reif, S.; Malka, R.; Shiller, D.A.; Zeilig, G. Self-Training to Improve UE Function at the Chronic Stage Post-Stroke: A Pilot Randomized Controlled Trial. *Disabil. Rehabil.* 2017, *39*, 1541–1548, doi:10.1080/09638288.2016.1239766.
